# Interventions addressing routine childhood immunization and its behavioral and social drivers

**DOI:** 10.3389/fpubh.2024.1364798

**Published:** 2024-06-19

**Authors:** Shradha S. Parsekar, Lalitha Vadrevu, Monica Jain, Shekhar Menon, Gunjan Taneja

**Affiliations:** ^1^International Initiative for Impact Evaluation (3ie), New Delhi, India; ^2^Bill & Melinda Gates Foundation, New Delhi, India

**Keywords:** behavior and social drivers, child immunization, interventions, low- and middle-income countries, zero-dose children

## Abstract

**Introduction:**

Despite the advances in vaccination, there are still several challenges in reaching millions of children in low- and middle-income countries (LMICs). In this review, we present an extensive summary of the various strategies used for improving routine immunization in LMICs to aid program implementers in designing vaccination interventions.

**Methods:**

Experimental and quasi-experimental impact evaluations conducted in LMICs evaluating the effectiveness of interventions in improving routine immunization of children aged 0–5 years or the intermediate outcomes were included from 3ie’s review of systematic reviews. Some additional impact evaluation studies published in recent years in select LMICs with large number of unvaccinated children were also included. Studies were coded to identify interventions and the barriers in the study context using the intervention framework developed in 3ie’s Evidence Gap Map and the WHO’s Behavioral and Social Drivers (BeSD) of vaccination framework, respectively. Qualitative analysis of the content was conducted to analyze the intervention strategies and the vaccination barriers that they addressed.

**Results and conclusion:**

One hundred and forty-two impact evaluations were included to summarize the interventions. To address attitudinal and knowledge related barriers to vaccination and to motivate caregivers, sensitization and educational programs, media campaigns, and monetary or non-monetary incentives to caregivers, that may or may not be conditional upon certain health behaviors, have been used across contexts. To improve knowledge of vaccination, its place, time, and schedule, automated voice messages and written or pictorial messages have been used as standalone or multicomponent strategies. Interventions used to improve service quality included training and education of health workers and providing monetary or non-monetary perks to them or sending reminders to them on different aspects of provision of vaccination services. Interventions like effective planning or outreach activities, follow-up of children, tracking of children that have missed vaccinations, pay-for-performance schemes and health system strengthening have also been used to improve service access and quality. Interventions aimed at mobilizing and collaborating with the community to impact social norms, attitudes, and empower communities to make health decisions have also been widely implemented.

## 1 Introduction

Despite the proven benefits of vaccination (1–3), a recent estimate shows that over 100 countries have stagnating or declining third dose of diphtheria-tetanus-pertussis (DTP3) coverage since 2019 (3, 4). An important factor in exacerbating this stagnation or decline is the COVID-19 pandemic and the resultant lockdown (5, 6). Even prior to 2019 and COVID-19, although there was a substantial improvement in immunization coverage, some of the countries struggled to improve or sustain coverage (2). Despite efforts to strengthen immunization services by national governments and international organizations (1, 3), childhood vaccination uptake differs across countries due to diversity in barriers to immunization, distribution of vaccination resources, and complexity in implementing the vaccination policies and programs (7). Identifying evidence on what interventions work for the uptake of childhood immunization, on whom, how, and why they work are important public health aims (7).

To improve the evidence base on community engagement interventions, International Initiative for Impact Evaluations (3ie) commissioned several evaluations of such interventions under the its Innovations in Increasing Immunization Evidence Program and prepared a series of synthesis products: Evidence Gap Map (EGM) (8) to map impact evaluations and systematic reviews of interventions for childhood immunization uptake, systematic review assessing effect of community engagement interventions on childhood immunization uptake (9), and a systematic review of systematic reviews (RoR) assessing effectiveness of different types of interventions for improving childhood immunization uptake in low- and middle-income countries (LMICs) (10). In the current review, we build on this existing work and use the intervention framework developed and described in 3ie EGM (8), to provide a compendium of interventions that have been implemented in LMICs to improve the routine child immunization outcomes.

We further analyze the barriers to vaccination uptake, classified using the WHO’s Behavioral and Social Drivers (BeSD) of Vaccination framework (11), that were potentially addressed through these interventions. The barriers to vaccination uptake which have been widely reported in the LMIC settings range from practical barriers such as time constraints, costs, health care access and caregivers’ knowledge to negative attitudes toward vaccination and fear of its adverse effects to social-cultural norms (12–16). A couple of recent review of reviews have systematically reviewed the evidence on interventions that have been effective in improving child vaccination uptake in LMICs. Besnier et al. (17) finds mixed effects of health promotion interventions but positive effects of interventions aiming to improve immunization communication, education, and social mobilization on vaccine uptake. The RoR (10) finds that caregiver-oriented interventions, especially those focusing on short-term sensitization and education campaigns as well as written messages to caregivers, are effective in improving vaccination uptake in LMICs. It also finds that community-oriented interventions are effective. Among the health system-oriented interventions it finds positive effects of home visits and mixed effects of pay-for-performance schemes.

While the existing literature provides evidence on barriers to vaccination uptake and to some extent on interventions to improve childhood vaccination uptake, it does not link the interventions to barriers being addressed in a structured manner, which we do in this paper. We also provide more information on key intervention features and implementation characteristics that are potentially useful for policy. Apart from standalone strategies, we also examine the different components of interventions that were co-implemented and whether the interventions were found to be successful in improving vaccination and/or its intermediate outcomes in the included evaluation studies.

## 2 Materials and methods

This is a summative review of the evidence from impact evaluations of interventions that measured outcomes pertaining to vaccination or its causal mechanisms, and their associated literature.

### 2.1 Inclusion criteria and source of evidence

We included primary studies cited in the 62 systematic reviews included in 3ie’s RoR (10). These systematic reviews were published until October 2021 assessing effectiveness of interventions on childhood vaccination. We prepared a list of 1,062 primary studies that were cited in 62 systematic reviews. These studies were screened for inclusion based on following inclusion criteria:

- Experimental and quasi-experimental studies establishing counterfactuals to determine the causal impact of an intervention in comparison to standard practice or alternative treatment.
- Studies conducted in LMICs, determined by its World Bank classification at the time an intervention was carried out.
- Studies assessing any intervention impacting routine immunization outcomes of children aged 0–5 years or the intermediate outcomes in the causal mechanisms (e.g., caregiver knowledge of childhood immunization).
- Studies published in English.

Further, to make this review more expansive, we also searched 3ie’s EGM^1^ (8) to include additional impact evaluations that were published between 2010 and 2020 and conducted in select LMICs with large number of unvaccinated children: India, Indonesia, and Pakistan from South and East Asia region; Ethiopia and Nigeria from Africa region; and Brazil and Guatemala from Latin America region. We ensured that these additional impact evaluation studies satisfied the above listed inclusion criteria and are not duplicates of the included primary studies from 3ie’s RoR.

### 2.2 Data extraction and synthesis

The details of the study components, context, geographies, and implementation process was extracted from the studies using the NVIVO software (Version 1.6.1). The Intervention Framework developed by 3ie (8) was used to classify the interventions and extract data. The framework broadly groups the interventions into caregiver-oriented, health system-oriented, community level, and policies and institutions. These broad themes are further divided into multiple level-two and -three interventions (Appendix 1 in Supplementary material). Furthermore, information pertaining to type of intervention, year of intervention, intervention objective, outcomes, country, contextual characteristics of the population such as economic or educational status, etc. were mapped. We also extracted data on the barriers addressed by these interventions and categorized them as per BeSD framework. The BeSD framework (9) was adapted for coding the information pertaining to barriers of childhood vaccination. The framework (9) divides the factors driving vaccine uptake into four broad drivers (1) thinking and feeling, (2) motivation, (3) social processes, and (4) practical constraints. Additionally, political climate, natural calamity, migration, and socio-economic characteristics that may affect the uptake of vaccination were captured. Codes pertaining to program or intervention characteristics, reasons for intervention success or failure, and impacts on drivers of vaccination were also created. Coding framework can be found in Appendix 2 in the Supplementary material.

Two researchers coded the data by dividing the included impact evaluations between them. Prior to this, an iterative process was followed to achieve coding consistency between the coders by piloting the coding framework on six studies. A detailed comparison table containing the researchers’ coding was created and discussed during a weekly meeting to identify the differences in coding and to standardize the definitions of the codes used. This process continued until there were minimal differences in the coding done by the two researchers. The coding framework and the definitions used for each of the codes were finalized at this stage. Redundant codes were deleted, memos or notes were added as per need, and the merged file was finalized for use in analysis.

We used a qualitative approach to synthesize the findings. We assessed the context, geographies, and program implementation process of the included studies. In addition, we examined the year-wise trends of interventions in included studies. We also segregated standalone and multicomponent strategies that addressed barriers of childhood immunization using BeSD framework. Within multicomponent strategies, each component was further classified as per the intervention framework developed in 3ie’s EGM (8). Within each intervention category, we briefly defined and described the intervention, provided the frequencies of identified studies and the context where they were conducted. For multi-component strategies, we also provided details of co-interventions. Further, within each intervention category, we listed the studies that had positive influence of intervention on at least one vaccination outcome. For this review, the impact evaluations that assessed multiple arms, each arm was counted as a separate intervention. We also provided a summary of the interventions for childhood immunization in LMIC contexts from this evidence base by linking the programs to the BeSD barriers.

## 3 Results

### 3.1 Description of included studies

**Figure 1:**
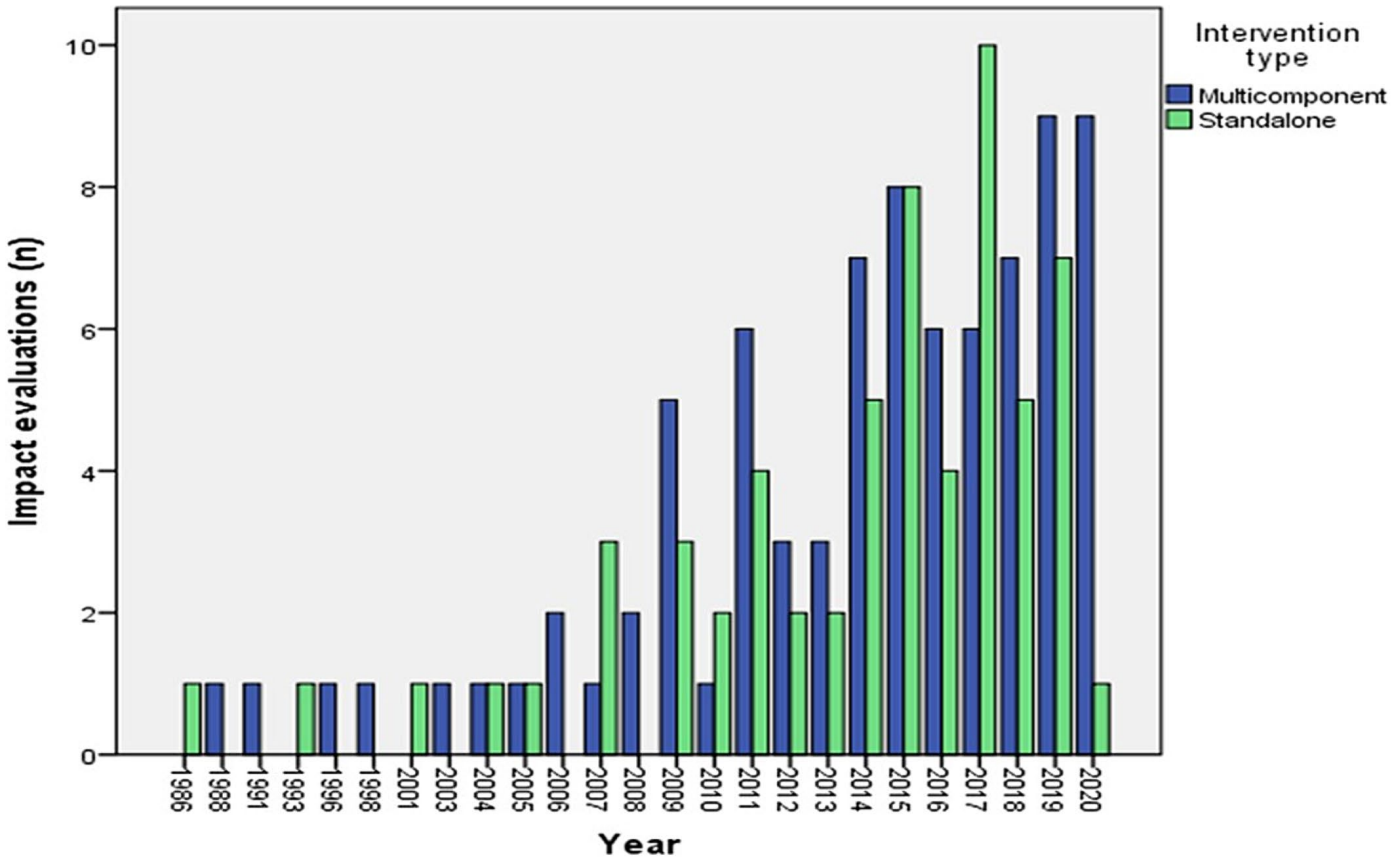
Year wise distribution of impact evaluations by intervention type.

Overall, 142 impact evaluations [116 from 3ie’s RoR (10) and 26 from 3ie’s EGM (8)] were included to synthesize the information on interventions and barriers. Included studies were mostly conducted in low-income settings within the countries. Some interventions targeted underserved hard-to-reach (HTR) regions having predominantly indigenous populations, slums, or street children. Many included studies were conducted in India (*n* = 42), followed by Nigeria (*n* = 13) (Supplementary Figure 3A). Some studies were conducted in conflict affected countries like Afghanistan, Burundi, Democratic Republic of Congo, Pakistan, Palestine, Nigeria, Ethiopia, and Guatemala as well as those prone to natural calamities such as floods, like India, Bangladesh, Indonesia, and Nigeria. Impact evaluations of interventions in populations with high degree of migration were from countries like India, Indonesia, and Nigeria. Details of the context and interventions of included impact evaluations can be found in Appendix 3 in the Supplementary material. Majority of studies (*n* = 111) were published between 2011 and 2020 and the oldest study was published in the year 1986 (18) (Figure 1).

### 3.2 Description of included interventions

**Figure 2:**
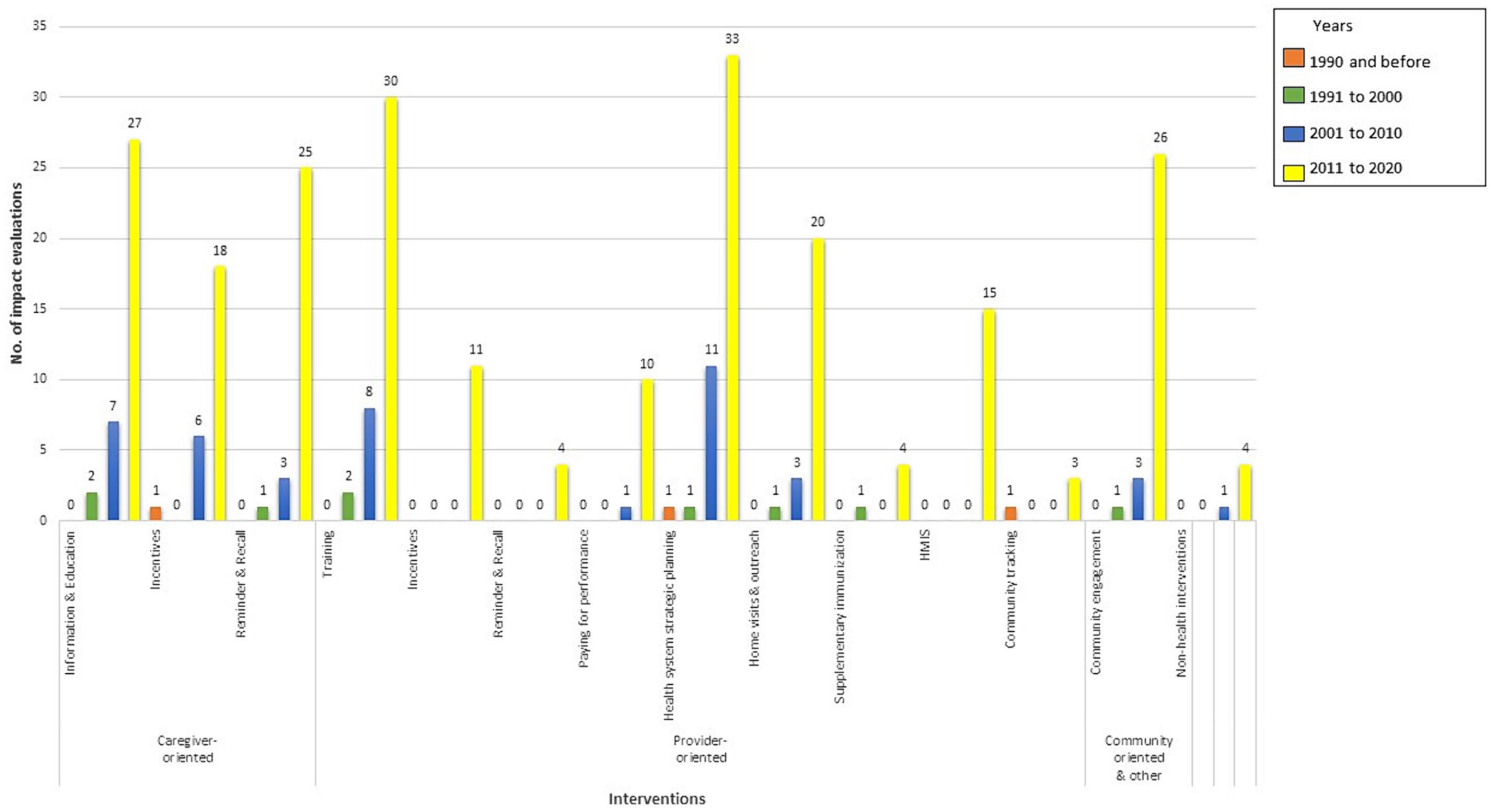
Intervention components (both standalone and multicomponent).

Evaluations of both standalone and multicomponent interventions have increased steadily since 2006 (Figure 1). In the 2001–2010 decade, the studies mostly focused on evaluating effectiveness of informing, educating, and incentivizing caregivers and training of health workers and health system strategic planning (Figure 2). In the 2011–2020 decade, there was an all-around increase in interventions oriented toward the caregiver, health system, and community. Among the caregiver-oriented interventions, there was a sharp jump in evaluations of reminder and recall interventions overlapping with increasing usage of mobile phones. Among the health system-oriented interventions, there was a big increase in evaluations of those training health workers, carrying out home visits and outreach as well as health system strategic planning. In addition, there were substantial number of evaluations of HMIS/Dashboard interventions. Furthermore, there was a sharp increase in evaluations of community engagement interventions.

The included impact evaluations investigated effects of multiple standalone or multicomponent strategies on immunization and its intermediate outcomes and one or more of the BeSD barriers to vaccination at various levels. We grouped these interventions based on the barriers that they address and described them below.

Strategies addressing barriers as per broad thematic areas of BeSD framework and their outcomes are listed in Table 1 (standalone strategies) and Appendix 4 (multicomponent strategies) in the Supplementary material. Table 2 is a summary of some of the multicomponent strategies that were co-implemented.

**Table 1:** Standalone strategies addressing behavioral and social drivers of vaccination and their impact on immunization.

Interventions	Barriers addressed				Intervention effect on immunization as reported by included studies	
	Think and feel	Social processes	Motivation	Practical barriers	Statistically significant positive effect	Insignificant effect
Short-term sensitization and education campaigns	Yes	–	Yes	Yes	*n* = 8 (19–26)	*n* = 1 (27)
Public information campaigns	Yes	–	Yes	-	*n* = 1 (28)	0
Material/monetary incentives for caregivers	–	–	Yes	Yes	*n* = 11 (29–39)	*n* = 5 (18, 40–43)
Automated voice messages to caregivers	Yes	–	Yes	Yes	*n* = 2 (44, 45)	*n* = 1 (46)
Written or pictorial messages (SMS, stickers, flyers etc.) to caregivers	Yes	–	Yes	Yes	*n* = 11 (37, 47–56)	*n* = 2 (46, 57)
Formal/Community health worker training and education	–	–	–	Yes	*n* = 3 (45, 58, 59)	0
National/sub-national immunization days	–	–	–	Yes	*n* = 1 (60)	0
Material/monetary incentives for health workers	–	–	–	Yes	*n* = 1 (61)	0
Non-monetary/material incentives	–	–	–	Yes	*n* = 2^ (62)	*n* = 1 (62)
Pay-for-performance schemes	–	–	–	Yes	*n* = 6 (63–68)	*n* = 8 (69–76)
Health system strategic planning	–	–	–	Yes	*n* = 2 (77, 78)	0
Changes to broader governance systems (beyond health systems)	–	–	–	Yes	*n* = 1 (79)	0
New HMIS/Dashboard systems (incl. Improved data collection)	–	–	–	Yes	*n* = 1 (80)	*n* = 1 (81)
Collaborating with whole community	–	Yes	–	–	*n* = 1 (82)	*n* = 1 (83)
Collaborating with selected community groups and networks	–	Yes	–	–	*n* = 1 (37)	0
Non-health/education infrastructure (e.g., electrification)	–	–	–	Yes	*n* = 4 (84–87)	0

In some of the included studies, it was not explicitly stated that the interventions addressed a particular barrier(s). We made reasonable assumptions on the barriers being addressed by considering the intervention components and causal pathways. For instance, pay-for-performance schemes can potentially improve performance of health workers thereby improving caregiver’s experiences of healthcare quality. Another example is prolonged exposure to media (public information campaigns) can potentially motivate caregivers. Furthermore, some of the strategies might also influence social processes, e.g., sensitization programs, but unless intervention included community mobilization activities or mentioned influencing the community, we did not mark them as intervention addressing social norms.^A study by Lee (62) assessed effect of three separate interventions (two of which were in Kenya and one in India).

**Table 2:** Co-implemented multicomponent strategies.

Interventions		Caregiver oriented interventions		Healthcare provider-oriented interventions			Community oriented interventions
Caregiver oriented interventions	Information or education strategies	Reminder/recall (88–90)	Incentives for CGs (91)	Incentives for HWs (92)	Home visits/Outreach to vulnerable populations (88, 92–98)	Health system strengthening and HW training (55, 89, 91, 92, 94, 96, 99–104)	Community mobilization (55, 88, 96–98, 101–107)
	Material/monetary incentives	Reminder/recall (57)	-	Reminder/recall and Incentives for HWs (108–111)	New HMIS/data collection systems or community tracking (57, 110)	Health system strengthening and HW training (91, 110–112)	-
	Mobile phone-based voice or text messages	Educational strategies (19, 88–90)	Incentives for CGs (57)	Reminder/recall (113–115)	New HMIS/data collection systems or community tracking (45, 80, 89, 113–118)	HW training (45, 89, 113)	-
Healthcare provider-oriented interventions	HW training and education	Reminder/recall and Educational strategies (45, 89, 94, 99–104, 119, 120)	Incentives for CGs (111)	Incentives for HWs (92, 111, 119, 121)	Home visits and other outreach activities (119, 120, 122–126)	Health system strengthening (100, 102, 119–121, 123–125, 127–137)	Community mobilization (101–104, 120, 124–126, 136, 137)
	Outreach to vulnerable populations	Educational strategies (92)	Incentives for CGs (138)	-	-	Health system strengthening and HW training (125, 126, 139–141)	Community mobilization (125, 126, 139–142)
	Home visits and other outreach activities	Educational strategies (88, 92–98, 119, 120)	-	Incentives for HW (119)	-	Health system strengthening and HW training (96, 119, 120, 122–124, 126, 143, 144)	Community mobilization (96–98, 120, 124, 126, 144–146)
	Material/monetary incentives for HWs	Educational strategies (92)	Incentives for CGs (108, 109)	-	New HMIS/data collection systems (121)	Health system strengthening and HW training (112, 121)	-
	Automated voice/written or pictorial messages to HWs	Reminder/recall and Educational strategies (113–115)	Incentives for CGs (110)	-	New HMIS/data collection systems or community tracking (113–115, 147)	Health system strengthening and HW training (110, 113)	-
	Health system strategic planning and other health system strengthening interventions	Reminder/recall and Educational strategies (92, 95, 100–103, 119, 120, 137)	Incentives for CGs (110, 148)	Incentives for HW (92, 119, 121)	Home visits and other outreach activities (119, 120, 123–125, 139–141)	HW training (100–103, 119–121, 123–125, 127–137, 149–152)	Community mobilization (101–103, 120, 124, 125, 136, 137, 139–141, 153–156)
	New HMIS/Dashboard systems	Reminder/recall (80, 113–118)	-	Reminder/recall (113–115)	Home visits and other outreach activities (143, 144)	Health system strengthening and HW training (113, 144, 147)	Community mobilization (144)
Community oriented interventions	Community mobilization	Reminder/recall and Educational strategies (88, 96–98, 101–107, 120, 137)	-	-	Home visits and other outreach activities (88, 96–98, 120, 124–126, 139–141, 144–146)	Health system strengthening and HW training (96, 101–104, 120, 124–126, 136, 137, 139–141, 144, 153–156)	-

CG, Caregivers; HW, Health workers. Study-wise details on intervention components, intervention addressing broad thematic area of BeSD framework and whether these were successful in improving immunization outcomes can be found in Appendix 4 in Supplementary material.

#### 3.2.1 Interventions addressing attitudes and knowledge about immunization

These interventions include informational and educational strategies such as short-term and sustained sensitization campaigns as well as public information campaigns (8).

##### 3.2.1.1 Short-term sensitization and education campaigns as a standalone strategy

Short-term sensitization and education campaigns are one-time interventions or those designed with a fixed end date in mind (8). There were nine studies using short-term sensitization campaigns as standalone strategies conducted in urban Indonesia (27), Pakistan (19, 20), China (21), and predominantly rural India (22–26). All of these studies, except one (27), found that the interventions were successful^2^ in improving immunization and intermediate outcomes such as maternal knowledge of immunization. These campaigns used video-assisted teaching packages, face-to-face health education and information, education and counseling strategies delivered via discussions, posters, leaflets, and flipcharts. The place of intervention was home, health facility, schools, places of worship or community centers. The interventions intended to improve knowledge about vaccine preventable diseases by focusing on benefits of vaccines, place of vaccination, and vaccination schedule. Interventions were either facilitated by physicians, medical students, trained researchers, community health facilitators or midwives. The focus of all interventions was on women during their pregnancy or childrearing, but one study also included school students (25).

##### 3.2.1.2 Short-term sensitization and education campaigns combined with other strategies

This review identified nine studies assessing short-term sensitization and education programs in combination with other strategies conducted in low-resource settings. Of these, six studies found that the interventions were successful in improving immunization and intermediate outcomes such as maternal knowledge of immunization outcomes (19, 88, 91, 93, 94, 105). The strategies co-implemented with short-term sensitization were media campaigns, mobile phone-based reminder/recall services, community engagement, and strategies to improve service quality by conducting outreach sessions like home visits and community health worker (CHW) trainings. For example, interventions in Ghana (93) and Nepal (99) included CHWs conducting short-term sensitization sessions during home visits. CHWs counseled the mothers regarding vaccination and reminded of the forthcoming appointments. The two studies from India conducted in the state of Uttar Pradesh combined short-term sensitization campaign with mass media campaigns, and reminder/recall services using technology. In one study, the intervention was delivered face-to-face in the community, and automated voice calls and a toll-free number were used to sensitize the community (88). While in another study, CHWs carried out video screening in the community (94). In a study conducted in Pakistan, center-based sessions were held to disseminate the information and motivate the caregivers about immunization, which were combined with reminder type immunization card for caregivers (19). While focus was on women, one intervention also included husbands and other family members, communities, and CHWs (94).

The other studies that had short-term sensitization intervention combined with other strategies (89, 91, 105, 157) are described under incentives and reminder/recalls to caregivers and community engagement strategies.

##### 3.2.1.3 Sustained sensitization and education campaigns combined with other strategies

Sensitization and education campaigns that were not designed with a fixed end date in mind were coded as sustained sensitization and education campaigns (8). Sustained sensitization and education of caregivers was used only in conjunction with other strategies. This review identified 16 studies that assessed these campaigns combined with other strategies. Of these, nine studies found that the interventions resulted in improved vaccination outcomes. The sustained sensitization strategies included health education by health professionals, trained peer educators, and women’s groups. The sustained sensitization strategies were combined with media campaigns, strategies for improving quality of services by conducting outreach vaccination services to vulnerable population, providing training to health workers, performance-based incentives, health check-ups, and tracking communities, and boosting political commitment and budgetary support. The examples of these programs were Growth and Development Monitoring Program in Colombia (158, 159), peer education intervention in Nigeria (95), and Muskan Ek Abhiyan in India (92).

##### 3.2.1.4 Public information campaigns as a standalone strategy

Public information campaigns refer to any mass media campaigns done through newspapers, social media, radio and television (8). A single study assessed the effect of social media platform, Twitter (now “X”), in Indonesia and demonstrated positive effect on knowledge and awareness about immunization. It was a nationwide dissemination campaign involving high-profile celebrities with millions of followers to retweet content focusing on improving knowledge and attitudes about immunization posted by the university students (28).

##### 3.2.1.5 Public information campaigns combined with other strategies

There were five multicomponent studies having one of the components as public information campaigns. Of these, all studies, except one (100), found that the interventions were successful in improving immunization outcomes. An example of public information campaigns was, a smiling sun campaign, a national health communication program in rural Bangladesh. It was launched to promote uptake of health services by disseminating key health messages through the logo posted at multiple clinic locations and through 26-episode television series, advertisements, radio spots, posters, newspapers, and local publicity (90).

#### 3.2.2 Behavior linked incentivized interventions for caregivers

Any incentives, such as material, monetary, or non-monetary perks that were used to nudge the mothers or caregivers to change their behavior toward immunization or health services were grouped under behavior linked incentives (8).

##### 3.2.2.1 Monetary or non-monetary incentives for caregivers as standalone strategy

Most of the incentivized programs used conditional cash transfers (CCT) to nudge mothers toward certain healthy behaviors or provided financial aid in accessing health services. The programs established conditionalities on utilizing and retaining school attendance and/or utilizing key health services for children (e.g., immunization) and pregnant women, with varying cash benefits offered. Conditional or unconditional cash transfers or food coupons as standalone strategies were implemented to motivate caregivers and offset opportunity and transport cost, thereby intended to improve maternal and child health (MCH), basic education and alleviate poverty. The brief description of the strategies by broad types is given below.

###### 3.2.2.1.1 Conditional monetary transfers at the household/individual level

This review identified 11 CCT programs, of which nine studies found that the intervention was successful in improving immunization outcomes, while two failed to improve immunization outcomes (40, 41). The Bolsa Familia Program in Brazil (29, 30, 42), the Program Keluarga Harapan (PKH or “Hopeful Family Program”) in Indonesia (31–33), Program Familias en Acción in Columbia (34), Progresa or Oportunidades Program in Mexico (35), Red de Protección Social in Nicaragua (35), and monetary program in Zimbabwe (41) transferred cash to poor households when they complied with conditions related to health and education. Similarly, the Subsidy Reinvestment and Empowerment Program (SURE-P) in Nigeria gave cash incentives to women residing in rural underserved areas, who sought maternal healthcare services such as the child births and post-natal care (PNC) at the facility (40). In India, caregivers were provided with a cash incentive for every girl child that was immunized and enrolled in the school (36).

###### 3.2.2.1.2 Village level performance incentives

Villages in Indonesia under the National Program for Community Empowerment (PNPM) Generasi received an annual block grant to be allocated to any activity that supported indicators of health and education service delivery. The study found that the program was successful in improving immunization outcomes (33).

###### 3.2.2.1.3 Conditional non-monetary transfers

This review identified four studies that provided non-monetary transfers to families upon receiving childhood immunization shots. Of these, three studies found that the interventions were successful in improving immunization outcomes while one study found that the intervention (18) was not successful. Incentives included mobile credit on the phone in India (37), food/medicine coupons or food supplements in Pakistan (38) and Nicaragua (18) and insecticide-treated bed nets in Malawi (39).

###### 3.2.2.1.4 Unconditional cash transfers

This review identified two unsuccessful unconditional cash transfers (UCTs) at individual or household level conducted in India (43) and Zimbabwe (41).

##### 3.2.2.2 Monetary or non-monetary incentives for caregivers combined with other strategies

This review identified 10 studies that implemented caregiver monetary or non-monetary incentive schemes as a multicomponent strategy conditional upon utilizing important health services. The other components combined with caregiver incentives were sensitization and education campaigns, mobile phone-based reminders to caregivers, improving healthcare service quality by introducing HMIS/Dashboard systems, training, incentivizing and reminding health workers and involving them in planning and monitoring, conducting outreach immunization services to vulnerable populations, tracking children for immunization, strengthening of peripheral health services that included setting up quality improvement teams and their training, and supplementary vaccination campaigns.

###### 3.2.2.2.1 Conditional monetary transfers for caregivers

This review identified three studies demonstrating improved immunization outcomes by implementing CCT programs. A monetary voucher program (programa de asignación familiar or family allowance program), in Honduras, provided monthly vouchers to households belonging to vulnerable groups with pregnant women or children under 3 years complying preventive health visits and if the child aged 6–12 years were enrolled in school. The program also strengthened peripheral health services by equipping with essential medicines and supply, upgrading health centers, and recruiting and training quality improvement teams (91). The Janani Suraksha Yojana, a CCT program in India, gave cash incentives to mothers that sought maternal health care services such as the institutional child births and post-natal care. The program also incentivized CHWs (namely ASHAs) who encouraged and assisted mothers in receiving health services (108, 109).

###### 3.2.2.2.2 Conditional non-monetary transfers for caregivers

There were seven studies that assessed non-monetary incentives in the form of materials such as insecticide treated bed nets, hygiene kits, lentils, phone talk time, and rewards. Of these, four studies found that the interventions were successful in improving immunization outcomes, while three studies (110, 111, 160) found that the interventions were not successful. The examples of conditional non-monetary transfers were, integration of insecticide treated bed nets with measles vaccination campaign in Madagascar (148). In Nicaragua, under Red de Protección Social progam, households received a food security transfer in alternate months based on mothers attending health education workshops held every other month and children under 5 years being brought for scheduled preventive health check-ups (160). In India, immunization camps in underserved areas were held monthly to improve outreach services along with providing small incentives such as lentils and thalis when the child was fully immunized (138). SMS reminders were provided to mothers regarding the next appointment, and they received the mobile phone credit upon getting their child vaccinated in another Indian study (57). The program in Bangladesh distributed vouchers to pregnant women entitling them to key antenatal and PNC services and cash stipends for transportation (161). A study in Ethiopia implemented “Protected Children” posters that incorporated a stamp system upon completion of each immunization visit. After fully immunizing the child, the mother received a completion symbol representing non-monetary reward. The intervention was also combined with tracking children, and poster acted as a reminders for health workers (110).

###### 3.2.2.2.3 Other strategies

Intended to improve socio-economic and nutritional status of destitute women and their children, the Rural Maintenance Program (RMP) was implemented in Bangladesh. Women received skill training in multiple activities required for income-generation and maintaining health and wellbeing. They also received small loans and micro-credits for income generation. The RMP was also linked with health centers to receive priority services. The program was not successful in improving immunization-related outcomes (157).

#### 3.2.3 Interventions addressing practical constraints faced by caregivers

Interventions that addressed practical constraints faced by caregivers in the uptake of immunization services (8). Interventions such as reminder or recalls to address lack of knowledge of immunization time, place and schedule, and strategies or programs to address availability, accessibility and quality of vaccination or health services are described in this section.

##### 3.2.3.1 Reminder or recall for caregivers as a standalone strategy

Reminder or recall strategies consist of reminding caregivers about upcoming immunization session, its place and time and may also contain motivational information. These strategies have been delivered via voice or text messages using mobile phone and/or paper-based printed material such as MCH handbook, flyer, or stickers.

###### 3.2.3.1.1 Automated voice messages to caregivers

This review identified two studies conducted in Mumbai, India (44) and Ibadan, Nigeria (45) that found automated educational and/or reminder voice messages to caregivers were effective in improving vaccination outcomes.

###### 3.2.3.1.2 Written or pictorial messages to caregivers

These include SMS, flyers, or stickers provided to caregivers. There were 11 studies that used written and pictorial messages as standalone strategy to remind mothers, mostly from low-income status, about upcoming immunization schedule and/or to disseminate information related to immunization. Of these, except one (57), all demonstrated positive effect of intervention on immunization-related outcomes. SMS-based reminders prior to scheduled immunization date or informational text were disseminated to mothers/caregivers in the studies conducted in Nigeria (47, 48), Guatemala (49), Pakistan (50), India (57), and Zimbabwe (51). Furthermore, in Indonesia, stickers were added to the home-based records to remind caregivers of their upcoming appointments (52). Similarly, CHWs in Pakistan, with the help of pictorial cards disseminated educational informational about vaccination benefits, place of vaccination, and importance of retaining immunization card (53). Studies in Ethiopia assessed the impact of reminder stickers (54) and in Bangladesh and Kenya (55, 56) evaluated MCH handbook. The handbook served as a tool for health information and education, reminders, and health record keeping.

###### 3.2.3.1.3 Automated voice and written or pictorial messages to caregivers

Two studies used combination of automated voice calls and text messages to caregivers. Of these, one demonstrated positive effect of intervention on immunization (37). The Phone Reminder for Immunization (PRIMM) program in Nigeria combined text messages with automated voice messages (46). While a study in India, in addition to reminders, disseminated information offsetting misconception regarding immunization (37).

###### 3.2.3.1.4 Other innovative ways of reminder or recall for caregivers

Innovative interventions such as the bracelets were used to track children and act as reminder for caregivers in Pakistan. Each time the child came for vaccination with the bracelet, a hole was punched in the symbol denoting the vaccine that was received on that visit and on being fully vaccinated the “star” symbol was punched. The study found that the intervention was successful in improving immunization outcomes (162).

##### 3.2.3.2 Reminder or recall for caregivers combined with other strategies

###### 3.2.3.2.1 MCH handbook combined with other strategies

Maternal and child health handbook is a booklet containing information about pregnancy, childbirth, and child health and serves as health record, e.g., vaccination. There were two studies that assessed the effect of MCH handbook on immunization outcomes that co-implemented sensitization campaigns and strategies to improve service quality such as health worker training. Of the two studies, one found that the intervention was successful which has been described under short-term sensitization strategies (19). The other intervention was implemented in Palestine, wherein MCH handbook was delivered to women during pregnancy registration at the clinic and the nurse counseled the women about the pregnancy and child care services. They also instructed women to retain the MCH card to keep vaccination and growth records. The nursing staff received 1-day training to use MCH handbook effectively (89).

###### 3.2.3.2.2 Mobile-phone based reminders to caregivers combined with other strategies

This review identified 11 studies that used multicomponent strategies having one of the components as voice or text message reminders to caregivers about upcoming vaccination schedule and/or educational information. Of these, eight interventions were found to be effective in improving immunization-related outcomes. The other strategies used in addition to text or voice calls were sensitization and educational campaigns, improving health service quality by providing training to health workers, reminder/recall phone-based messages for health workers, implementing new HMIS or dashboard systems, capacity building for existing system, home visits and tracking children. For example, in Nigeria, one arm of the study assessed combination of cell phone reminder calls and training of health providers. Calls were made 1 and 2 days before the scheduled immunization date and repeat calls were made if the child missed immunization (45). The details of other multicomponent studies (57, 80, 113–118) that had voice or text message reminders to caregivers are described under sections new HMIS or dashboard system and interventions addressing attitude and knowledge about vaccination (88, 90).

##### 3.2.3.3 Strategies to improve access to vaccination center

Strategies that focused on improving access to immunization services such as road improvement programs, and outreach activities are discussed in this section. In India, two studies assessed the impact of Prime Minister’s Rural Road Program (*Rural Gram Sadak Yojana*). The studies found that the construction of roads would lead to increased accessibility to healthcare facility by caregivers (84) and improved health care supply, increased household income, increased awareness, and improved social interaction in the village (85).

In addition to improving access to roads, outreach to vulnerable groups and migrant populations was also used in some interventions. In Bangladesh and India, outreach activities were undertaken as a part of supplementary mass vaccination campaign to influence delivery of vaccines to the community (60, 163). Another study implemented community-based intervention in underserved urban migrant community living in slums of Punjab, India, through immunization outreach clinics to improve access. This was supported by the community nominated guardians to carry out post-immunization surveillance (142). All these studies demonstrated positive effect of intervention in improving immunization-related outcomes.

There were other multicomponent strategies focusing on outreach and home visits; these are described under interventions addressing health service quality and community engagement.

#### 3.2.4 Interventions to improve health systems

##### 3.2.4.1 New HMIS/dashboard systems (incl. improved data collection)

This review identified 14 studies that tested mobile-based applications to be used by health workers for data collection or tracking children. Most of these studies employed multicomponent strategies that also included reminder voice or text messages to caregivers and/or health workers, capacity building of health workers to use these applications and outreach activities. Of the 14 studies, six found that the intervention was effective in improving immunization-related outcomes.

Mobile application-based studies conducted in low resource settings of India (ImTeCHO), Bangladesh and China used reminder messages to caregivers and health workers. The m-health application was also used to improve data collection and tracking children (113, 114, 117). In ImTeCHO and ReMinD projects in India, CHWs (ASHAs) received daily work assignments and decisional support on the mobile application. ASHAs also used phone to show video clips to mothers during their home visits (81, 113). Similarly, a study in Ethiopia introduced m-health application for health extension workers (HEWs) to register pregnant mothers and children, receive reminder messages about scheduled antenatal or vaccination date, planning resources and tracking mothers and children who missed scheduled appointments (147). The RapidSMS program of Rwanda tracked mothers and children till 2-year of age and sent SMS reminders to both the providers and the mothers regarding follow-up visits (115). Whereas, in Guatemala under the Coverage extension program (Programa de Extensión de Cobertura) using dashboard, a list of mothers and children eligible for preventive health services was generated and shared with health workers. Health workers then visited the households to remind caregivers about the scheduled appointment (143). In a study conducted in Côte d’Ivoire, through mobile web service platform mothers received either SMS or voice call reminders about the upcoming immunization schedule and two more reminders for non-compliance (118). Another example is the Khushi Baby program, a digital pendant worn by child and voice reminder platform introduced in underserved areas of Udaipur, India. Automated voice reminder calls were sent to caregivers about the upcoming immunization camps using dashboard. Khushi baby app was used by the health workers to scan the pendant to update real-time summary statistics such as immunization records and document other necessary health system related information. The dashboard also helped CHWs in planning and decision-making (80, 116).

Prinja et al. (81) and one arm of Nagar et al. (80) assessed the effect of New HMIS or Dashboard systems as a single component, while the rest were combined with other interventions.

##### 3.2.4.2 Strategies to enhance capacity and/or performance of health workers

###### 3.2.4.2.1 Health worker training as standalone strategy

This review identified three studies which found employing health worker training as effective standalone strategy for improving immunization outcomes. Two studies conducted in Ibadan, Nigeria using a standard manual provided refresher training to primary healthcare immunization providers (*viz.* nurses, midwives, community health officers, and community HEWs) (45, 58). In Indonesia, on-the-job training was provided by the senior nurses to less-experienced or underperforming immunization nurses. Training was about maintaining quality of vaccines, practical advice, instruction on operating the information system and discussion of ways to increase coverage. Additionally, trainers received recognition, a paid trip to the host health center, and formal credit toward advancement (59).

###### 3.2.4.2.2 Health worker training combined with other strategies

This review identified 36 multicomponent interventions that recruited and/or trained human resources. Of these, majority, i.e., 22 studies found that the interventions were successful in improving immunization outcomes, while 14 studies found that the interventions were not successful in improving immunization outcomes (89, 99–101, 111, 119, 120, 122, 127–130, 149, 164). The training of healthcare providers was often combined with sustained sensitization programs, media campaigns, improving health service quality by improving outreach activities including home visits, supplementary immunization activities, nonmonetary incentives, pay-for-performance, supervising staff, involving health workers in planning and monitoring, upgrading the infrastructure, and new HMIS system and community engagement strategies. Training was done either as a part of a national health system strengthening initiatives or as an intervention to improve quality of childcare. For example, in India formal health workers and CHWs received trainings to strengthen their skills and knowledge, and encourage them to mobilize and motivate women for service utilization including immunization (111, 119, 122, 149, 150). Similar examples come from the continuous quality improvement initiatives (151) and Essential Services for Health project in Ethiopia (121), Midwife service scheme (100) and addressing missed opportunities for immunization (131) in Nigeria, the government and non-government collaborative project in Rwanda (164) and the program in Senegal (127). A study in Georgia, implemented a set of activities that included development of supportive supervision guidelines for district immunization managers, district-level training in continuous supportive supervision, monitoring and evaluation of performance, and funding for district center for public health to carry out the package of interventions (132).

There were other strategies that included training of frontline health workers, which are described under health system strategic planning and community engagement strategies.

###### 3.2.4.2.3 Standalone monetary incentives for health workers

In Pakistan, a standalone strategy on time preferences to customize for polio vaccinators was successfully used to motivate health workers (61).

###### 3.2.4.2.4 Standalone non-monetary incentives for health workers

This review identified three interventions conducted in Zambia and India wherein non-monetary or material incentives were provided to health workers. Two of these were found to be effective in improving immunization-related outcomes. In Zambia, intervention included promotion prospects and career advancement (e.g., nurse, clinical officer etc.) for newly recruited Community Health Assistants (CHWs). Based on performance, CHWs were eligible for in-service training for career advancement freely by the government. In the second experiment, nationwide 1-year training program was provided in Zambia for CHWs to be regularized in services. In the third experiment, a self-tracking app delivered information that made efforts more intrinsically rewarding for CHWs (ASHA) workers in India. ASHAs tracked their performance through data visualization (or audio version) and was dependent on their own submission via phone-based reporting tool (62).

###### 3.2.4.2.5 Monetary incentives for health workers combined with other strategies

Monetary incentives that are conditional on performance of the provider were used to incentivize health workers and other providers to improve health service delivery. Studies included five multicomponent strategies on monetary incentives for providers (92, 108, 109, 111, 161). These strategies were found in combination with sustained sensitization campaigns, incentivizing caregivers and improving health service quality by providing training and non-monetary incentives to health workers. Of the five studies, all studies, except one (111), found that the intervention was successful in improving immunization-related outcomes.

###### 3.2.4.2.6 Non-monetary incentives for health workers combined with other strategies

These include two studies in which material or non-material incentives were provided to motivate the healthcare providers to improve their performance. These were combined with service-level quality improvement strategies such as training and incentivizing health workers. Of these, one study found that the intervention improved vaccination outcomes (121). The other study was Team-based Goals and Incentives Initiative in India, in which CHWs (ASHAs and auxiliary nurse midwives) got quarterly incentives that typically consisted of stoves, casseroles, storage containers, or other similar household items. An additional so-called bumper prize (a pressure cooker) and a certificate was given at the end of the year to CHWs in those sub centers that successfully met their targets in all four quarters (165).

###### 3.2.4.2.7 Performance-based financing as standalone strategy

The performance-based incentives refer to monetary transfers to health center/facility for reaching a certain service delivery target(s) and may include some proportion of transfers going to the providers. This review identified 14 studies that assessed such strategies. Of these, six studies found that the strategies were successful in improving immunization-related outcomes (63–67, 69). These studies have been conducted in Haiti (66), Afghanistan (70), Rwanda (71–73), Burundi (63), Democratic Republic of Congo (69, 74, 75), Cameroon (65, 68), Tanzania (67, 76), and Zambia (64).

*Performance-based financing as a multicomponent strategy* was implemented in several contexts. For example, in India it was implemented as part of Integrated Management of Childhood Illnesses (IMCI) program (119), which found insignificant effect on immunization outcomes. Another example was the Nigeria State Health Investment Project to improve quality of care. Funds transferred to the health facility were used for operational costs and healthcare provider bonus. Results indicated that the immunization-related outcomes improved (112).

##### 3.2.4.3 Health system strategic planning

###### 3.2.4.3.1 Health system strategic planning as standalone strategies

Health system strategic planning refers to a broad array of strategies intended to strengthen health system. These include initiatives at the national or sub-national level to develop plans and governance structures designed to improve vaccination or other MCH services. Additionally, these include interventions that improve the human resource availability, strategies, policies, and plans in existing health governance and delivery structures that may or may not be directly related to immunization services (8). For example, as a part of Family Health Program in Brazil, provision of comprehensive care was done through multi-professional teams. These teams were responsible for promotion, prevention, and care of mothers and children in a designated area (77). In Mali, intermittent preventive treatment of malaria at the time of routine vaccination was introduced irrespective of presence of malaria symptoms or infection in children (78). Both interventions demonstrated improved immunization outcomes.

###### 3.2.4.3.2 Health system strategic planning as multicomponent strategies

This review identified 37 studies that implemented health system strategic planning components; of these 16 studies found that the intervention demonstrated improved immunization-related outcomes. The strategies identified from included studies were mostly large-scale programs intended to improve service delivery by addressing shortage of human resources and conducting outreach activities. The strategies included provision of comprehensive care, recruiting and training of CHWs, volunteers, religious leaders and other front-line workers, measures to improve quality of services, upgradation of services, and equipping with essential drugs and supply.

Many of the interventions using health system strategic planning were large-scale programs for engaging community and recruiting and training new cadres of CHWs to depute them to peripheral health centers to strengthen promotion and preventive health activities. These CHWs conducted home visits to provide essential services at the household-level, track children for immunization and encourage the community for uptake of health services (e.g., immunization). CHWs were paid honorarium for their services. To support the CHWs, peripheral health centers were created or upgraded and equipped with drugs and supply to manage basic health services. These programs were implemented in Bangladesh—IMCI (120), Ethiopia—Community-based Health Services Extension Program (123), Zambia—Community health assistant program (133), and South Africa—Ward-based outreach teams (134). Some of these programs in addition to above listed strategies also included other intervention components. For example, in Bangladesh, other stakeholders such as religious leaders, health workers and theater artists were trained to convey health messages to address IMCI issues (120). In India, under Integrated Child Development Services Scheme local village women, namely Anganwadi workers (AWWs) with the help of helpers delivered the health, nutrition and pre-school services (153). In China’s Gasu province, health sector reform by reducing the cost of care at the point of care was implemented. The strategies were also implemented to protect and finance old commune-based insurance scheme, and introduction of health expense safety nets for the poorest to cover MCH services (130).

The other components listed under health system strategic planning included introduction of auto-disabled syringes, reducing cost of care, intermittent malaria treatment, supply and replenishment of HIV testing commodities and cognitive behavioral therapy. For instance, a quality improvement approach namely, Client-Oriented, Provider Efficient Services was implemented to strengthen health system and support IMCI efforts in Guinea and Kenya. It was a participatory, problem-solving approach by trained health provider; wherein providers identified problem areas, suggested solutions and took actions. External facilitators oriented district supervisors and intervention site managers, who then supervised the intervention (135). A study in Madagascar introduced auto-disabled (AD) syringes in national immunization program. Additionally, training of health workers on national immunization policies, open vaccine vial, and AD syringe usage was provided (129). In Pakistan, in a program called Thinking health, a cognitive behavior therapy-based intervention was implemented for depressed perinatal women delivered by trained lady health workers. The intervention was guided by techniques of active listening, collaboration with the family, guided discovery, and homework (152). In Zambia, the intervention included integrating early infant HIV diagnosis with the expanded program on immunization. Supply and replenishment of HIV testing commodities and a sensitization meeting with facility staff was held by district health officials (128).

The other multicomponent studies that included health system strategic planning strategies are described under interventions addressing attitudes and knowledge of immunization, community mobilization and training of health workers.

###### 3.2.4.3.3 Other strategies

The Ethiopian government introduced Fiscal decentralization project, wherein more powers were given to district and urban administrators in order to improve service delivery (86). Another example was a rural electrification program (*Jyotigram Yojana*) in Gujarat, India. The motivation of the project was that by improving electricity to the rural areas, access to information via television and internet would improve and households would see time endowment effect. Electricity would also improve cold chain management at the facility level (87). Furthermore, in the state of Andhra Pradesh, India, women’s political reservation was assessed. The intervention was theorized on the basis that female leaders would favor policies useful to human capital, and they would increase investment in education and health care (e.g., vaccination) (79). All these three studies demonstrated positive effect of interventions on immunization-related outcomes.

#### 3.2.5 Intervention strategies used to mobilize communities and influence social norms

Interventions aimed at mobilizing and collaborating with the community to impact social norms, attitudes, and empower communities to make health decisions have been widely implemented. These strategies either collaborated with the whole community or select community groups such as self-help groups, village committees, religious groups, or women’s organizations. Most of these strategies were multicomponent in nature.

##### 3.2.5.1 Community engagement or mobilization as standalone strategy

This review identified three studies that engaged community (whole or select groups) as a standalone strategy. All the three studies were conducted in India. Of these, two studies found that the interventions were successful, while one study found that the intervention (83) was not successful in improving immunization-related outcomes. In one study, Village Health and Sanitation Committees (VHSCs) were tested as part of the National Health Mission. VHSCs focused on political agency wherein community members were consulted for resource allocation at the village level. VHSCs developed village health plans and managed an untied fund to enable local planning and action. They organized health-promotion activities and mobilized pregnant women and children to access maternal and health care services (82). Another intervention was a participatory approach in Mumbai slums to identify problems, potential solutions, planning, implementing, and monitoring potential solutions and information sharing (83). Furthermore, one of the arms of a study conducted in Haryana, carried out a gossip seed (social network) experiment by engaging selected community members. The study identified key community members having wide community network (or gossip seeds), were trusted (trust seeds), were trusted with wide network (trusted gossip seeds) or any individual (random seeds) to spread information related to immunization (37).

##### 3.2.5.2 Community engagement or mobilization as multicomponent strategy

This review identified 27 multicomponent strategies that included components of collaboration with select community groups or the whole community. Of these, 21 studies found that the intervention improved immunization-related outcomes. Interventions included building community groups or leveraging existing groups to increase awareness and decision-making among caregivers. Collaboration strategies also included mobilizing the community to identify community resources and needs to demand health care. The strategies also engaged community to strengthen peripheral health service provision. These strategies were combined with sustained sensitization activities, media campaigns, interventions to improve service quality such as capacity building of health care workers, health system strengthening, introducing new HMIS or dashboard systems and improving outreach activities.

The community engagement strategies included identifying and training suitable community members/committees from different positions and background which could be newly selected members or members from recognized community groups/association, health providers and policymakers from a designated area/community. These committees discussed important health topics (e.g., barriers to vaccination or healthcare access) using participatory approaches and took actions to mitigate them. The committee cultivated accountability, shared the necessary information, used the resources responsibly and helped in improving governance. The health workers, research team, community themselves or NGO monitored and evaluated the progress made by community members/ committees. The program strategies also included dissemination of information to a larger community through sensitization and media campaigns or with the help of local leaders. The examples of community engagement strategies are Evidence-based discussion intervention in Pakistan (105), Bottom-up community engagement intervention in Ghana (154), Health governance intervention in Afghanistan (155), Community Health Strategy in Kenya as a part of National Health Sector Strategic Plan (136), and Community-based monitoring intervention in Uganda (156). Furthermore, in Bangladesh, participatory women’s groups (106) and existing rural self-help organizations (137) were trained and engaged to promote health in the community. In India, examples of community engagement initiatives were Mahila Samakhya, a women’s empowerment program to set up women’s group in underserved regions of Bihar belonging to socially and economically marginalized backgrounds (107) and social accountability interventions in Uttar Pradesh (145). A study in Nigeria, engaged and trained traditional and religious leaders to promote the active participation and positive engagement of communities in immunization. Leaders were also engaged in maintaining cold chain (96).

The multicomponent programs intended to improve targeted services in some of the underserved regions also engaged community. These programs identified and trained CHWs to provide essential services in underserved areas by carrying out outreach activities and home visits. The program also strengthened the peripheral services and ensured supply of vaccines and logistics. The local or religious leaders supported the CHWs or co-managed the multicomponent programs by initiating dialogs, mobilizing community, and outreach activities. The examples were Community Health Strategy in Kenya (124, 136), a multicomponent strategy using m-Reach platform in Uganda (125), and in Nigeria, sensitization and mobilization of mothers (97) and using trained community volunteers and CHWs as part of IMCI program (102). The other programs that included community engagement strategies alongside strengthening of peripheral health services and outreach activities were conducted in other contexts also. For example, in India a community resource center strategy in Mumbai slums (146), Stimulate, Appreciate, Learn, and Transfer community engagement approach project (98) and Reaching Every District strategy in Assam (144) and Community Mobilization and Integrated Planning (Bachpan Program) in underserved tribal regions of Madhya Pradesh (103). Similarly, in Guatemala, to address transport and informational barrier, under Coverage Extension Program (Programa de Extensión de Cobertura), provision of services in underserved community was made by contracting out of primary health services to non-governmental organizations (NGOs). Through mobile teams, the NGOs provided the health services to HTR areas at least once a month. Additionally, community facilitators recruited and supervised local staff (e.g., midwives), who helped in managing logistics of service provision (139–141). Furthermore, as part of Maternal Neonatal Child Survival program in Bangladesh, health services provision and sensitization were made at the community and facility by collaborating with whole community (101). A study in conflict-affected regions of Pakistan facing polio resistance engaged community members and political leaders and intensified immunization activities by conducting outreach camps with strict monitoring and supervision (126). In mountainous regions of Pakistan, community were engaged by forming community health committee as part of community-based perinatal and newborn preventive care package (104).

## 4 Conclusion and implications for research and practice

The summative review article serves as a brief on the wide range of strategies used to improve immunization outcomes and links them with the BeSD barriers and sub-barriers being potentially addressed. Interventions included in this review are majorly focused on improving knowledge on immunization benefits, awareness regarding the schedule of vaccination, motivation of caregivers, and quality of health services. Interventions focusing on improving attitudinal and knowledge related barriers to vaccination, used sensitization programs and public campaigns alone or in combination with other strategies. Many of the interventions on conditional monetary or non-monetary incentives were government programs and were used for motivating caregivers into adopting healthy behaviors. Many interventions used m-health interventions for reminding caregivers regarding upcoming vaccinations and the place and time of vaccination. These strategies were also used to disseminate information related to the benefits of vaccinations and motivate caregivers. To address access related barriers, outreach services, home visits and road improvements strategies were implemented. Interventions used to improve service quality used training and education of frontline health workers, as well as providing monetary or non-monetary perks to encourage or remind them to provide vaccination services, pay-for-performance schemes and health system strategic planning. Most of these strategies were combined with others to improve service delivery. Many of these interventions were part of government programs that focused on existing resources, e.g., ASHAs in India, and health extension workers in Nigeria and Ethiopia. Most of the interventions on community mobilization were multi-component interventions that leveraged community to address the attitudinal and service delivery aspects of immunization.

It is to be noted that while we provide information on whether the program improved vaccination outcomes by studies, we cannot draw inferences on the effectiveness of a specific strategy as this evidence has not been critically appraised. Also, the review did not intend to assess the cost-effectiveness of the interventions as typically only a handful of evaluations provide the required information (9). Another limitation of this study is that while we summarize a large evidence base of 142 impact evaluations, because of resource constraints, we could only include primary studies which were cited in included reviews in the RoR (10) and some recent ones which were available in 3ie’s EGM (8) from select countries with large number of unvaccinated children. In addition, the information within the studies did not allow us to distinguish barriers to vaccine uptake by the vaccination status of children. For example, we could not assess if barriers for zero-dose children are different from those for drop-outs. Also, the findings were dependent on the study context and the degree of reporting in the manuscript. Not all studies clearly mentioned the barriers that were addressed by the intervention. Therefore, we made assumptions and identified the barriers in the study context from the objectives of the intervention strategies and causal pathways. For instance, pay-for-performance schemes can potentially improve performance of health workers thereby improving caregiver’s experiences of healthcare quality and hence the vaccine uptake.

While we acknowledge the limitations mentioned above, we also recognize that there is a dearth of high-quality systematic reviews that rigorously synthesize evidence on effectiveness of interventions by their types (10). In addition, the current literature on interventions provides only a very high-level overview of interventions (17, 166, 167). In such a situation, the current review fills an important learning gap by providing detailed summary of strategies from 142 impact evaluations. Considerable information on the intervention components, activities, and implementation features has been extracted and synthesized in a way that is easy for use by program managers and policy makers. It allows them to be informed on a range of strategies which have been tested to address a barrier along with the program contexts in which they have been tested. This review also separates the standalone strategies from those which have multi-components. For the latter it also provides information on the other major components as well as the broad barriers they potentially aimed to address. This allows the program managers to know the combinations in which multi-components interventions have been tested and their contexts. To summarize, this review allows the program managers and policy makers who are exploring various strategies to improve vaccination uptake (a) to know which strategies have been tested and in which combinations, (b) to know the context in which they have been tested, and (c) to easily identify and explore the studies as their references are provided.

This review also highlights critical areas for future policy relevant research design. Many of the national and regional level interventions included in this review had multi-components with complex impact pathways, but only a few included a detailed theory of change (ToC). To understand how and why an intervention impacts an important barrier subsequently influencing uptake of vaccination, a well-developed ToC with carefully laid out impact pathways is helpful. To evaluate these pathways and key underlying assumptions in a ToC, a mixed-methods evaluation design with an integrated process evaluation is important. However, only a few studies had this design and were mostly 3ie funded (37, 94, 96, 98, 116, 125). In addition, it is not only important to assess what works but also at what cost, therefore, future studies could explore this important research gap. Furthermore, better reporting in future research studies on various barriers in the study contexts as well as interventions components will be useful for identifying the link between the two. The article hopes to serve as a quick reckoner for program managers in LMIC context.

## Author contributions

SP: Conceptualization, Data curation, Formal analysis, Investigation, Methodology, Visualization, Writing – original draft, Writing – review & editing. LV: Conceptualization, Data curation, Formal analysis, Investigation, Methodology, Visualization, Writing – original draft, Writing – review & editing. MJ: Conceptualization, Funding acquisition, Project administration, Resources, Supervision, Writing – review & editing. SM: Writing – review & editing. GT: Writing – review & editing.

## Footnotes

^1^The EGM includes 226 completed and 26 ongoing impact evaluations, and 58 completed and one ongoing systematic reviews. The search of EGM was comprehensively done and was last updated in May 2020.

^2^We have considered an intervention to be effective/successful as long as the evaluation study found that it improved at least one immunization-related outcome, irrespective of whether it was primary or secondary outcome.
