# Supplementary material for: Interventions addressing routine childhood immunization and its behavioral and social drivers
Source: Front Public Health. 2024 Jun 19;12:1364798. doi: 10.3389/fpubh.2024.1364798 (PMC11223502; doi:10.3389/fpubh.2024.1364798)
Supplement: Supplementary file 1 [file Table_1.DOCX]

**Supplementary data file**

**Table of Contents**

[**Appendix 1: Intervention-outcome framework and definitions** 2](#_Toc148711318)

[**Appendix 2: Coding framework** 11](#_Toc148711319)

[**Appendix 3: Details of context, and intervention and its impact** 21](#_Toc148711320)

[**Figure 3A: Countries of included impact evaluations** 70](#_Toc148711321)

[**Appendix 4: Multicomponent interventions addressing behavioral and social drivers of vaccination and their impact on immunization** 71](#_Toc148711322)

# **Appendix 1: Intervention-outcome framework and definitions**

(Source: Engelbert et al., 2022)

**Interventions**

| Intervention | | | Definition/guidance |
| --- | --- | --- | --- |
| A. Caregiver-oriented | AA. Information & education | AA1. Sustained sensitization and education campaigns | Sustained interventions (i.e., those that are *not* designed with a fixed end date in mind) that provide targeted caregivers with information about immunisation and its importance, the vaccination schedule, or where and how to access immunisation services. For example, village health and nutrition days (VHNDs) in India in which health education and counselling services are provided to pregnant women and mothers of young children on a regular basis. |
|  |  | AA2. Short-term sensitization and education campaigns^[[1]](#footnote-1)^ | One-off interventions (i.e., those designed with a fixed end date in mind) that provide targeted caregivers with information about immunisation and its importance, the vaccination schedule, or where and how to access immunisation services. |
|  |  | AA3. Public information campaigns | Mass media campaigns through newspapers, radio and TV which provide caregivers with information about immunisation and its importance, the vaccination schedule, or where and how to access immunisation services. Because of the nature of communication mediums they cannot be targeted to a specific audience. |
|  | AB. Incentives & motivation | AB1. Material/ monetary incentives for caregivers | Interventions that incentivise caregivers to vaccinate through items with monetary value. This could be cash transfers or material goods like food or home goods. |
|  |  | AB2. Non-material incentives for caregivers | Interventions that seek to motivate caregivers to vaccinate through non-material incentives like social recognition. Unlike reminder messages, interventions in this category should seek to *create* or *strengthen* a desire to vaccinate, rather than activating a standing intention to vaccinate. |
|  |  | AB3. Automated voice messages to caregivers | Use of automatically-generated voice messages (usually delivered to a mobile phone) that remind caregivers about upcoming vaccinations, provide them information on place and time of vaccination and encourage them to vaccinate. While these messages may contain some motivational component (e.g., stressing the importance of vaccination in addition to reminding caregivers about an upcoming appointment), voice messages should be categorised only here and not also under “non-material incentives”, unless they rely on a substantive motivational factor like social recognition. |
|  |  | AB4. Written or pictorial messages (SMS, stickers, flyers etc.) to caregivers | Use of written messages/pictorial that remind caregivers about upcoming vaccinations, provide them information on place and time of vaccination and encourage them to vaccinate. While these messages may contain some motivational component (e.g., stressing the importance of vaccination in addition to reminding caregivers about an upcoming appointment), messages should be categorised only here and not also under “non-material incentives”, unless they rely on a substantive motivational factor like social recognition. |
|  |  | AB5. Changes to health system user fees | Any change to the monetary costs to users for accessing the health system. This can include introduction or elimination of fees at the point of service, or pre-payment or insurance schemes. |

| Intervention | | | Definition/guidance |
| --- | --- | --- | --- |
| B. Health system-oriented | BA.  Education & training | BA1. Formal health worker training and education | Programmes that train or educate formal health workers (FHWs). FHWs are typically vaccinators (and they tend to provide/prescribe medication or administer tests such as recording blood glucose level, etc.). (The only likely exception to this would be oral polio vaccination (OPV), especially supplementary polio campaigns, where CHWs or community volunteers may be enlisted to administer the vaccination.) |
|  |  | BA2. Community health worker training and education | Programmes that train or educate community health workers (CHWs). CHWs are defined as “paraprofessionals or lay individuals with an in-depth understanding of the community culture and language, have received standardised job-related training of a shorter duration than health professionals, and their primary goal is to provide culturally appropriate health services to the community.”^[1]^ |
|  | BB.  Planning, implementation, monitoring | BB1. Formal health worker involvement in planning & monitoring | Interventions that give FHWs substantive roles in creating plans/strategies to deliver vaccination services and/or monitor vaccination coverage in the community. |
|  |  | BB2. Community health worker involvement in planning & monitoring | Interventions that give CHWs substantive roles in creating plans/strategies to deliver vaccination services and/or monitor vaccination coverage in the community. |
|  |  | BB3. Paper-based tracking | Paper-based systems (e.g., logbooks) used by health workers to keep track of children in the community who are due for upcoming vaccinations or have not received scheduled vaccinations. Note that if a study merely mentions the existence of logbooks, that is not sufficient to code it as this intervention. The logbooks must be specifically used for tracking upcoming and missed vaccinations. |
|  |  | BB4. Promoting outreach to vaccine-hesitant groups | Outreach to groups that, because of religious, cultural, or other reasons, are suspicious of vaccination or have specific fears about it (e.g., that vaccinations cause infertility or spread disease). |
|  |  | BB5. Outreach to vulnerable populations (hard-to-reach, SES, caste, etc.) | Outreach to groups that are vulnerable in a way that affects their access to vaccination services. They may be in hard-to-reach geographical areas, have low socioeconomic status (including wealth and education), or be from groups marginalised based on caste, ethnicity, etc. This also includes interventions that set up temporary and mobile clinics to deliver vaccines, to make vaccination services more accessible. |
|  |  | BB6. Outreach to migrant populations | Outreach to populations who have migrated temporarily or seasonally because of cultural or employment reasons. |
|  |  | BB7. Home visits | Use of visits to caregivers’ homes by health workers. This includes both visits to encourage caregivers to vaccinate their children, and visits to deliver vaccines, provided they are *targeted* visits to specific households for routine immunization. General door-to-door campaigns to deliver specific vaccines go under BC1. |
|  |  | BB8. Campaigns to vaccinate refugee populations | Interventions that make a special effort to vaccinate populations which have been displaced temporarily or permanently because of conflict, war or famine. |
|  | BC.  Supplementary Immunisation Activities | BC1. National/sub-national immunisation days | Supplementary immunisation activities (SIA) are mass immunisation campaigns which complement routine immunisation activities whereby a vaccine is taken simultaneously to many residents of a community within a defined short space of time. They have generally been conducted for polio and measles. They may be called national or sub-national immunisation days. They may happen through booth days or door to door vaccination campaigns. On a booth day a large number of fixed site booths are set up throughout the target area for children to be brought to receive a specific vaccine like polio. In door to door campaigns vaccination teams go door to door to every house, checking each child under five to see if they have received the specific vaccine, and if they have not that vaccination is provided. |
|  | BD.  Incentives & motivation | BD1. Material/monetary incentives for health workers | Interventions that incentivise formal or community health workers to deliver vaccination services through items with monetary value. This could be cash transfers or material goods like food or home goods. |
|  |  | BD2. Non-material incentives for health workers | Interventions that use non-material incentives like social recognition to incentivise formal or community health workers to deliver vaccination services. |
|  |  | BD3. Automated voice messages to health workers | Use of automatically-generated voice messages (usually delivered to a mobile phone) that remind health workers about upcoming vaccinations for community members, provide them information to help plan their work, and encourage them to conduct outreach to those community members. |
|  |  | BD4. Written or pictorial messages (SMS, stickers, flyers etc.) to health workers | Use of written/pictorial messages that remind health workers about upcoming vaccinations for community members, provide them information to help plan their work, and encourage them to conduct outreach to those community members. |
|  |  | BD5. Pay-for-performance schemes | Schemes whereby health centres or districts receive funding based on their performance in delivering health services (e.g., they receive a given amount of funding for each child vaccinated or each antenatal care visit completed). Also known as “results-based financing”. |
|  | BE.  Infrastructure | BE1. Building & upgrading health clinics | Projects that build new permanent health clinics, or provide physical upgrades to existing clinics. |
|  |  | BE2. Cold chain infrastructure improvements | Interventions that improve the ability of health systems to maintain vaccine cold chains. |
|  | BF.  Health system governance, policies and financing | BF1. Health system strategic planning | Initiatives at the national or sub-national level to develop plans and governance structures designed to improve vaccination services. This also includes interventions that improve the human resource availability, strategies, policies and plans in existing health governance and delivery structures that may or may not be directly related to immunisation services. |
|  |  | BF2. Vaccination guidelines | Changes to official national or subnational guidelines about when and how vaccinations should be administered. This includes studies comparing two different approaches to administering vaccines (e.g., one measles dose vs. two), which could be made into guidelines. |
|  |  | BF3. Changes to broader governance systems (beyond health systems) | Interventions that modify general governance systems not directly related to health. An example would be a policy dictating a certain level of representation for women in local or national governing bodies. |
|  |  | BF4. Health system financing | Interventions that increase the national or sub-national financing of health or specifically for vaccination in absolute terms or as a proportion of GDP. This also includes results- or performance-based financing. |
|  | BG. Technology & mHealth | BG1. New HMIS/Dashboard systems (incl. improved data collection) | New digital tools and systems designed to improve health system capacity to monitor and deliver vaccination services. This includes apps for mobile phones or tablets, as well as desktop-based software and setting up online dashboards. A common type of intervention in this category is giving health workers tablets with an app that allows them to register vaccination information (and for other health services) for community members, track their vaccination schedules and provide them readily-accessible information about vaccination. |
|  |  | BG2. Capacity building (e.g., training) for existing systems | Initiatives to train people working in the health system (including frontline health workers and administrative personnel) to improve their ability to use existing digital tools and systems more effectively. |
| C. Other community member-oriented | CA. Other community member-oriented | CA1. Faith-based outreach/outreach using local leaders | Interventions that enlist influential community members (often religious or other traditional leaders) to promote vaccination in the community. |
| D. Community-level | DA. Communication & dialogue | DA1. Collaborating with whole community | Interventions that involve or plan to involve ALL community members beyond health workers in various aspects of the intervention, such as developing plans and solutions to improve immunisation outcomes in the community. |
|  |  | DA2. Collaborating with selected community groups and networks | Interventions that involve *selected* groups or networks of community members beyond health workers (other than the traditional or religious leaders who fall under CA1) in developing plans and solutions to improve immunisation outcomes in the community. This includes interventions focusing on mother’s groups, father clubs, self-help groups, etc. |
|  | DB. Tracking & registering | DB1. Community tracking and registering | Interventions that involve community members beyond health workers (e.g., caregivers or any other community members other than the traditional leaders who fall under CA1) in registering children with the health system so their vaccination status can be tracked, and/or tracking which children are due for vaccinations. |
| E. Policies and institutions | EA. Education policy and infrastructure | EA1. Education policy and infrastructure | Policy interventions that affect education levels of people in a country or region. An example would be an intervention that makes education mandatory for a particular population where it had previously been optional. |
|  | EB. Non-health/ education infrastructure | EB1. Non-health/ education infrastructure (e.g., electrification) | General improvements in physical infrastructure beyond the health system. This may include electrification, roads, sanitation improvements, etc. |

**Outcomes**

| Outcome | | | | | | | | | | | Definition/guidance | | |
| --- | --- | --- | --- | --- | --- | --- | --- | --- | --- | --- | --- | --- | --- |
| H. Behavioural, social and practical barriers faced by caregivers and communities | | | HAA. Thinking and feeling (attitudes, confidence) | | | | HAA1. Knowledge about immunisation | | | | Caregivers’ knowledge about immunisation in general (i.e., its purpose and role in preventing disease) | | |
|  |  |  |  |  |  |  | HAA2. Attitudes about immunisation | | | | Caregivers’ attitudes towards immunisation in general (i.e., whether they view it favourably or unfavourably or have high or low confidence in its efficacy) | | |
|  |  |  |  |  |  |  | HAA3. Attitudes about health providers | | | | Caregivers’ attitudes about health providers in general (i.e., whether they generally trust health providers to provide high-quality and appropriate care) | | |
|  |  |  | HAB. Social processes | | | | HAB1. Community norms | | | | Community-level attitudes and beliefs about immunisation, including whether there is social pressure to vaccinate or not vaccinate. This can be measured either objectively through aggregating community-level responses or subjectively by soliciting individual community members’ beliefs about the norms in their community. This includes attitudes and beliefs about immunization of key influencers in the community like traditional or religious leaders. | | |
|  |  |  |  |  |  |  | HAB2. Household norms & decision-making | | | | Norms and practices determining who in a household (e.g., mother, father, mother-in-law) provides input to decisions about whether to vaccinate, and how much decision-making power individual household members have. This also covers attitudes towards immunisation of household members other than the primary caregiver. | | |
|  |  |  | HAC. Readiness to vaccinate | | | | HAC1. Readiness to vaccinate | | | | Caregivers’ motivation, intention and plan to vaccinate their children. Note this is more specific than general attitudes towards immunisation covered under HAA2 | | |
|  |  |  |  |  |  |  | HAC2. Reasons for not vaccinating | | | | Caregivers’ stated reasons for not vaccinating children. This may include factors such as convenience (which would also be coded under “perceived convenience of vaccination”), but only use this code if the factors mentioned are specifically framed as reasons for not vaccinating. Also, only use this code when the study measures *effects* of the intervention on this outcome. If the study gathers data on reasons for not vaccinating but does not provide an effect size for this as an outcome, use the cross-cutting theme but not this outcome code. | | |
|  | HAD. Practical factors | | | | | | HAD1. Awareness of place, time, schedule for vaccination | | | | Caregivers’ knowledge about when and where they should go for vaccinations. | |  |
|  |  |  |  |  |  |  | HAD2. Actual cost of vaccinating | | | | Actual cost of vaccinating the child, including vaccine cost, transportation cost, loss of wage/income due to missed work, and providing gifts/unofficial payments to the health providers | |  |
|  |  |  |  |  |  |  | HAD3. Perceived convenience of vaccination | | | | Subjective measures (i.e., caregivers’ beliefs) of the convenience of taking the child for vaccination, such as opportunity costs of vaccinating a child (e.g., not able to care for a younger child), long lines at health clinic, and inconvenient day/time of vaccination. | |  |
|  |  |  |  |  |  |  | HAD4. Experience and satisfaction with health services | | | | The actual experience of health services in the last visit such as duration of waiting time, availability of vaccine or vaccinator, and behaviour of the health staff (respect, rudeness). This also includes a level of satisfaction with the health services, professionals and facilities. | |  |
|  |  |  |  |  |  |  | HAD5. Vaccination health card availability/ retention | | | | Measures of whether caregivers possess vaccination health cards provided by the health system, and/or whether caregivers can show the vaccination health card. | |  |
|  |  |  |  |  |  |  | HAD6. Perception of vaccination side effects | | | | Caregivers’ perceptions of the likelihood and severity of side effects from vaccination, and their knowledge of how to recognise and treat normal side effects. | |  |
| I. Delivery of vaccination services | | | IA. Health workforce | | IAA. Comm. health workers | | | IAA1. Community HW motivation, capacity & performance | | | | Any measure of CHWs’ capacity to deliver quality and timely vaccination services. |  |
|  |  |  |  |  |  |  |  | IAA2. Supply of CHWs | | | | The total availability of CHW services in the community, taking into account both the number of CHWs and the time they have available. |  |
|  |  |  |  |  | IAB. Vaccinators | | | IAB1. Formal HW supply | | | | The total availability of FHW services in the community, taking into account both the number of FHWs and the time they have available. |  |
|  |  |  |  |  |  |  |  | IAB2. Availability of HWs at vacc. point of service | | | | Whether vaccinators are present at vaccination point of service (e.g., health clinic) when vaccination services are supposed to be offered. This includes measures of health worker “absenteeism”. This is different from perception or experience of health workers from caregivers’ point of view. The source of information for this outcome can be administrative data or survey of health facilities and staff. |  |
|  |  |  |  |  |  |  |  | IAB3. Formal HW motivation, capacity & performance | | | | Any measure of FHWs’ capacity to deliver quality and timely vaccination services, or of their performance in doing so. |  |
|  |  |  |  |  | IAC. Administrators | | | IAC1. Admin staffing | | | | The number of staff in administrative posts in the health system (i.e., those not directly involved in health service provision). |  |
|  |  |  |  |  |  |  |  | IAC2. Capacity of health admin. responsible for vaccination | | | | The knowledge, skills, and motivation of staff in administrative posts in the health system, including leadership positions. |  |
|  |  |  | IBA. Health information systems | | | | | IBA1. Immunisation data collection (quality, completeness) | | | | The health system’s capacity for and success in collecting data about vaccination coverage and service quality for regular monitoring and accountability. |  |
|  |  |  |  |  |  |  |  | IBA2. Defaulter tracing | | | | The health system’s capacity for and success in identifying vaccination “defaulters” (i.e., those whose children receive early vaccine doses but do not return for subsequent vaccinations). |  |
|  |  |  |  |  |  |  |  | IBA3. Supply chain management | | | | The health system’s capacity to monitor the supply of vaccines at points of service, ensure reliable supply chains, and avoid supply “bottlenecks”. |  |
|  |  |  |  |  |  |  |  | IBA4. Immunisation data availability/ transparency | | | | How easy it is for stakeholders within and beyond the health system to access data about vaccination service quality, coverage and timeliness. |  |
|  |  |  | ICA. Vaccine availability | | | | | ICA1. Stockouts | | | | The frequency and duration of incidents when vaccines are out of stock at points of service, or when vaccines are in stock but cannot be administered to children who are brought in (e.g., because health workers are instructed not to open a new vial if there are not enough children to receive all doses in the vial, meaning that some of the vaccine would go to waste). Source of this information is administrative data and/or survey of health facilities and staff |  |
|  |  |  |  |  |  |  |  | ICA2. Quality of cold chain infrastructure | | | | The availability, quality and upkeep of physical equipment and place for cold chain storage of vaccines |  |
|  |  |  | IDA. Resources | | | | | IDA1. National or sub-national vaccine financing | | | | Change in financial resources for national or sub-national vaccination programmes, policies or strategies. |  |
| JAA. Vaccination coverage | | | | JAA01. Full routine immunization for children | | | | | Binary measure of whether or not children have received all routine vaccinations for the relevant country or region. | | | |  |
|  |  |  |  | JAA02. BCG | | | | | Binary measure of whether or not children have received the BCG vaccine. This may be measured by checking whether children have a BCG vaccination scar. | | | |  |
|  |  |  |  | JAA03. DPT1 | | | | | Binary measure of whether children have received first dose of the DPT/penta vaccine. If study does not specify which doses were included in the outcome measures (i.e., the outcome is just “DPT/pentavalent vaccination” then apply this code unless it is clear they are talking about full DPT/penta vaccination, in which case code it as JAA05: DPT3. | | | |  |
|  |  |  |  | JAA04. DPT2 | | | | | Binary measure of whether or not children have received the second dose of the DPT or pentavalent vaccine | | | |  |
|  |  |  |  | JAA05. DPT3 | | | | | Binary measure of whether or not children have received the third dose of the DPT or pentavalent vaccine. If the study does not specifically say “DPT3” (or “pentavalent 3”), but refers to “complete DPT/penta vaccination”, then use this code. | | | |  |
|  |  |  |  | JAA06. OPV0 | | | | | Binary measure of whether children have received 1^st^ dose of oral polio vaccine (recommended for administration at birth). | | | |  |
|  |  |  |  | JAA07. OPV1 | | | | | Binary measure of whether children have received 2^nd^ dose of the oral polio vaccine (recommended for administration at 6 weeks). | | | |  |
|  |  |  |  | JAA08. OPV2 | | | | | Binary measure of whether children have received the 3^rd^ dose of the oral polio vaccine (recommended for administration at 10 weeks). | | | |  |
|  |  |  |  | JAA09. OPV3 | | | | | Binary measure of whether children have received the 4^th^ and final dose of the oral polio vaccine (recommended for administration at 14 weeks). | | | |  |
|  |  |  |  | JAA10. IPV | | | | | Binary measure of whether children have received inactivated polio vaccine, given as injection. Countries differ in their guidelines/practices regarding IPV, so, please note the number of doses and age(s) when administered. | | | |  |
|  |  |  |  | JAA11. Measles | | | | | Binary measure of whether or not children have received the measles vaccine | | | |  |
|  |  |  |  | JAA12. No vs. partial routine immunization | | | | | Proportion of children who receive at least one vaccination versus those who are completely unvaccinated. | | | |  |
|  |  |  |  | JAA13. Vacc. timeliness | | | | | Proportion of vaccinations delivered on time according to the recommended schedule, vs those that are delivered late. | | | |  |
|  |  |  |  | JAA14. Drop out rate for multi-dose vaccines | | | | | Proportion of children who fail to receive the complete course of a multi-dose vaccine (DPT/penta, OPV, or in some cases measles) after receiving the first dose. | | | |  |
|  |  |  |  | JAA15. Vaccination coverage (unspecified) | | | | | Use this code if the evaluation or SR refers to impacts on routine vaccination coverage for children, but without specifying which vaccines. | | | |  |
| KAA. Health outcomes | | | | KAA1. Childhood morbidity | | | | | Incidence of vaccine-preventable diseases or symptoms associated with those diseases (e.g., diarrhoea) among children under 5. | | | |  |
|  |  |  |  | KAA2. Neonatal/Infant/ Child Mortality | | | | | Incidence of mortality among children below five years from all causes. Neonatal mortality refers to death of a live-born baby within the first 28 days of life. *Infant mortality* is the death of young children under the age of 1. Child mortality, refers to the mortality of children under the age of five. This category includes all-cause mortality. | | | |  |

[1] From Olaniran, A., Smith, H., Unkels, R., Bar-Zeev, S., & van den Broek, N. (2017). Who is a community health worker? – A systematic review of definitions. *Global Health Action*, *10*(1), 1272223.<https://doi.org/10.1080/16549716.2017.1272223>.

# **Appendix 2: Coding framework**

| Name | Description |
| --- | --- |
| 1. Thinking and feeling (confidence and attitude) |  |
| a. Attitudes about immunization (confidence in vaccine benefits and or or safety) | Caregivers’ attitudes about or confidence in immunization and its benefits. |
| i. Favorable attitudes |  |
| Any other favorable attitude towards immunization | such as ‘’want’’ or ‘’intend’’ to vaccinate the child. |
| Confidence in vaccine benefits or importance of vaccination | Select this code if the paper states that caregivers have knowledge or awareness of importance of immunization or express confidence in vaccine benefits. Examples: “Vaccines for children are safe and effective.” OR “I know MMR vaccine protects the child against measles.” OR “Vaccines do a good job in preventing the diseases they are intended to prevent.” OR “Childhood vaccines are important for my child’s health.”, etc. |
| Favorable religious or personal beliefs | Select this code if the paper states that caregivers religious or personal beliefs encourage immunization of children. Examples: “Vaccines are compatible with my religious beliefs.” OR “I believe vaccines should be compulsory or vaccine mandates are important.” OR “To protect public health, we should follow government guidelines about vaccines.”, etc. |
| No fear of side effects | Select this code if the paper states that fear of side effects were not observed among caregivers |
| ii. Unfavorable attitudes |  |
| Any other unfavorable attitude about vaccination |  |
| Fear of vaccination and or or its side effects | Select this code if the paper states that fear of side effects were observed among caregivers. Examples: “I fear that my child will fall sick after vaccination.” OR “My older child fell sick after vaccination so I did not get my younger one vaccinated.” OR “My child got fever and diarrhea post vaccination and now I fear taking my child for other vaccinations.” OR “Vaccinations are not safe.” OR “vaccinations cause disease to my child.” Or “vaccination leads to autism.” Or “I worry about the unknown effects of vaccines in the future.” OR “I fear needles, hence I do not get my child vaccinated.”, etc. |
| No awareness or knowledge of importance of vaccination | Select this code if the paper states that caregivers have no knowledge or awareness of importance of immunization. |
| Unfavorable religious or personal beliefs | Select this code if the paper states that caregivers religious or personal beliefs are unfavorable for immunization of children. Examples: “Authorities promote vaccination for financial gain, not for people’s health.” OR “Vaccines are not compatible with my religious beliefs.” OR “Vaccines are against the force of nature.” OR “Vaccines are impure.” OR “Vaccines contain other organisms/ are not vegetarian.”, OR “I did not think it was important to get my child vaccinated because she is a girl.” OR “Vaccine causes infertility.” OR “Vaccinations are painful for my child as they are too many shots in one go” OR “Natural immunity would better protect my child against disease.” “We also got this disease while growing up and it posed no long-term harm to our health and wellbeing.” OR “They give too many vaccination to my child in one go.” OR “Vaccines contain dangerous ingredients” |
| b. Attitudes about health providers (confidence in provider) | Caregivers’ attitudes about health providers in general (i.e., whether they generally trust health providers to provide high-quality and appropriate care) |
| Lack of trust or confidence in health provider | Select this code if the paper states that caregivers have unfavorable attitude towards health providers. Examples: “I do not trust the health worker to provide reliable information about the benefits of vaccination / other medical conditions.” OR “I don’t trust my health worker because she is employed by the government and they don’t care for the wellbeing of people.” Or “Other people in the community listened to this health worker and their children fell sick, hence, I do not trust her.” OR “my health provider pressurized me into getting my older child vaccinated and they suffered from side effects. Hence, I do not trust their advice.”, etc. |
| Trust or confidence in health providers | Select this code if the paper states that caregivers have favorable attitude towards health providers. Examples: “The health worker only recommends vaccination / medicine to improve the health of our children and family.” OR “The health worker addressed all my concerns regarding the health of my child.” OR “I was comfortable getting my child vaccinated because the health worker explained the possible side effects and how to manage them.” OR “The information I receive about vaccines from the vaccine program is reliable and trustworthy.” OR “Generally I do what my doctor or health care provider recommends about vaccines for my child/children.”, etc. |
| 2. Social processes |  |
| a. Community norms | Community-level attitudes and beliefs about immunization, including whether there is social pressure or encouragement to vaccinate or not vaccinate. This includes attitudes and beliefs about immunization of key influencers in the community like traditional or religious leaders. |
| i. Neighbors or friends |  |
| Neighbors or friends do not support immunization | Select this code if the paper states that caregivers have neighbors or friends who discourage immunization of children. |
| Neighbors or friends support immunization | Select this code if the paper states that caregivers have neighbors or friends who encourage immunization of children. |
| ii. Traditional or religious leaders (TRL) or other influencers such as local political leaders |  |
| TRL or other influences support immunization | Select this code if the paper states that the community under study has TRL or other influential people who encourage immunization of children. |
| TRL or other influences who do not support immunization | Select this code if the paper states that the community under study has TRL or other influential people who discourage immunization of children. |
| iii. Public mechanisms to encourage vaccination | An example could be that in a community during town halls, caregivers of unvaccinated children are publicly called out to socially pressuring them to vaccinate their children. If such public mechanisms exist, select this code. |
| b. Household norms and decision making | Select this code if the paper mentions norms and practices determining who in a household (e.g., mother, father, mother-in-law) provides input to decisions about whether to vaccinate, and how much decision-making power individual household members have. This also covers attitudes towards immunization of household members other than the primary caregiver. |
| c. Local or community health providers engagement with caregiver | This includes local medical doctors and health workers. Select this code if the paper mentions explicit statements about interaction between the caregivers and the local health providers. |
| Negative engagement | Examples: “My local or community health provider has not recommended that I get my child vaccinated.” OR “My community health worker did not follow up with me on missed vaccination appointment.” |
| Positive engagement | Examples: “My local or community health provider has recommended that I get my child vaccinated.” OR “My community health worker followed up with me on missed vaccination appointment.” |
| d. Any other social process or norm |  |
| 3. Practical constraints |  |
| a. Awareness of place and time for vaccination | Caregivers’ knowledge about when and where they should go for vaccinations. |
| Aware | Select this code if the paper states that caregivers know the place and time of vaccination |
| Partially aware | Select this code is that paper states that caregivers partially know the place and time for vaccination |
| Unaware | Select this code is that paper states that caregivers do not know the place and time for vaccination |
| b. Awareness of vaccination schedule | Caregivers’ knowledge about the vaccination schedule. |
| Aware | Select this code if the paper states that caregivers know schedule of vaccinations. |
| Partially aware | Select this code is that paper states that caregivers partially know the vaccination schedule |
| Unaware | Select this code is that paper states that caregivers do not know the vaccination schedule |
| c. Cost | Cost of vaccinating the child which could fall into any of the following subcodes |
| i. Actual cost of vaccination |  |
| Barrier_Absent | Select this code if the paper states that caregivers did not have to pay for the actual cost of vaccination |
| Barrier_Present | Select this code if the paper states that caregivers had to pay for the actual cost of vaccination |
| ii. Cost of transport |  |
| Barrier_Absent | Select this code if the paper states that caregivers did not have to pay for transportation to reach the vaccination center |
| Barrier_Present | Select this code if the paper states that caregivers had to pay for transportation to reach the vaccination center |
| iii. Opportunity cost |  |
| Barrier_Absent | Select this code if the paper mentions that caregivers’ opportunity cost or perceived convenience did not pose as barriers to vaccination. |
| Barrier_Present | Select this code if the paper mentions caregivers’ opportunity cost or perceived convenience as barriers to vaccination. This could include statements such as ‘caregivers had to give up a day’s wage to get their child vaccinated’ or ‘It was inconvenient for the caregiver to take the child for vaccination as they had to finish household chores. |
| iv. Unofficial payments to health workers or others |  |
| Barrier_Absent | Select this code if the paper states that caregivers did not have to pay/bribe HWs to get their child vaccinated |
| Barrier_Present | Select this code if the paper states that caregivers had to pay/bribe HWs to get their child vaccinated |
| v. Out of pocket expenditure for treating AEFI |  |
| Barrier_Absent | Select this code if the paper states that caregivers did not have to pay to treat AEFI. |
| Barrier_Present | Select this code if the paper states that caregivers had to pay out of their pocket to treat AEFIs |
| vi. Any other costs related to vaccinations |  |
| Barrier_Absent | Select this code if the paper states that caregivers did not have to incur any other cost to get their child vaccinated |
| Barrier_Present | Select this code if the paper states that caregivers had to incur any other cost to get their child vaccinated |
| d. Ease of access or logistics | Absence or presence of barriers related to access of services. These could include distance or logistics related challenges. |
| i. Distance from the point of vaccination |  |
| Barrier_Absent | Select this code if the paper states that caregivers did not have to face distance or logistics related challenges to get their child vaccinated. |
| Barrier_Present | Select this code if the paper states that caregivers faced distance or logistics related challenges to get their child vaccinated. |
| ii. Inconvenient health or vaccination clinic times |  |
| Barrier_Absent | Select this code if the paper states that caregivers did not have to face this challenge |
| Barrier_Present | Select this code if the paper states that caregivers faced this challenge. |
| iii. Inability to reproduce vaccination or health card at the point of vaccination | Examples include vaccination misplaced or damaged by caregiver or caregiver forgets to get the card for the vaccination appointment. |
| Barrier_Absent | Select this code if the paper states that caregivers did not have to face this challenge. |
| Barrier_Present | Select this code if the paper states that caregivers faced this challenge. |
| iv. Any other challenge |  |
| e. Experience and satisfaction with health services | The actual experience of health services in the last visit such as duration of waiting time, availability of vaccine or vaccinator, and behavior of the health staff (respect, rudeness). This also includes level of satisfaction with the health services, professionals and facilities. |
| i. Long wait times |  |
| Barrier_Absent | Select this code if the paper states that caregivers were not satisfied with the health services provided to them/their children |
| Barrier_Present | Select this code if the paper states that caregivers were satisfied with the health services provided to them/their children |
| ii. Health care provider availability at the point of vaccination |  |
| Available | Select this code if the paper states that HWs were available at the point of vaccination |
| Unavailable | Select this code if the paper states that caregivers were not available. |
| iii. Stockouts |  |
| Barrier_Absent | Select this code if the paper states that caregivers did not have to face vaccine stockouts. |
| Barrier_Present | Select this code if the paper states that they were refused vaccination due to stockout. |
| iv. Health provider refusal to open vaccine vial |  |
| Barrier_Absent | Select this code if the paper states that HWs had did not have a problem in opening a new vial for vaccination even if there was a chance of wastage. |
| Barrier_Present | Select this code if the paper states that HWs refuse to open a new vial of vaccine to avoid wastage. |
| v. Health provider behavior |  |
| Disrespectful | Select this code if the paper states that caregivers thought the HWs were rude while providing vaccination services |
| Respectful | Select this code if the paper states that caregivers found HWs to be respectful/kind during the delivery of vaccination services |
| vi. Quality of health facility, services or infrastructure |  |
| Adequate | Select this code if the paper states that the caregivers found the physical infrastructure of the health facility or the services adequate. |
| Inadequate | Select this code if that paper states that caregivers found the physical infrastructure of the health facility or the services inadequate. Example: “Vaccinator/ health worker cannot vaccinate accurately or dependably.” |
| vii. Any other factor |  |
| f. Any other practical constraint |  |
| 4. Service delivery constraints (from health system perspective) |  |
| a. Health worker attitudes, motivation, capacity and performance | Any measure of frontline health workers’ (FHW) or community health workers’ (CHWs) capacity to deliver quality and timely vaccination services. |
| i. Training or support | Whether delivery of services is impacted by presence or lack of training, supportive supervision, etc. |
| Barrier_Absent | Select this code if the paper states that health workers did not face challenges related to their motivation, capacity to carry out their duties and their performance |
| Barrier_Present | Select this code if the paper states that health workers mentioned challenges related to their intrinsic motivation, capacity to carry out their duties and their performance. |
| ii. Payment delays, low wage or non-payment of wages | Whether health workers’ motivation or capacity to deliver services is hampered by low wages or non-payment of salaries. |
| Barrier_Absent | Select this code if the paper states that health workers did not face challenges related to low wages or non-payment of salaries |
| Barrier_Present | Select this code if the paper states that health workers mentioned challenges related to low wages or non-payment of salaries |
| iii. Health worker attitudes about vaccination |  |
| Favorable attitudes |  |
| Unfavorable attitudes |  |
| iv. Others | Any other reasons specified |
| b. Supply of health workers | The availability of FHW or CHW services in the community, taking into account both the number of HWs and the time they have available |
| Barrier_Absent | Select this code if the paper states that health workers availability/ supply was not constrained |
| Barrier_Present | Select this code if the paper states that health workers availability/ supply was constrained |
| c. Stockouts | The frequency and duration of incidents when vaccines are out of stock at points of service, or when vaccines are in stock but cannot be administered to children who are brought in (e.g., because health workers are instructed not to open a new vial if there are not enough children to receive all doses in the vial, meaning that some of the vaccine would go to waste). Source of this information is administrative data and/or survey of health facilities and staff. |
| Barrier_Absent | Select this code if the paper states that stockouts were not a challenge. |
| Barrier_Present | Select this code if the paper states that stockouts were a frequent challenge. |
| d. Quality of infrastructure | The availability, quality and upkeep of physical equipment and place for cold chain storage of vaccines |
| Barrier_Absent | Select this code if the paper states that quality of health services or health infrastructure did not pose a challenge |
| Barrier_Present | Select this code if the paper states that quality of health services or health infrastructure was poor |
| e. Quality of services or service delivery |  |
| Barrier_Absent | Select this code if the paper states that quality of health services or health infrastructure did not pose a challenge |
| Barrier_Present | Select this code if the paper states that quality of health services was poor. This is includes not maintaining health records or not checking records of children who come for other health services, i.e., increasing chances of missed opportunities for vaccination. Other examples could include health worker not administering multiple injectable vaccinations as per the immunization schedule. |
| 5. Other contextual factors |  |
| a. Political climate | Discussion of the political context. Example: Political unrest made it difficult to access health services |
| b. Natural calamity or geographical inaccessibility | Discussion of any natural calamity impacting the study area. Example: floods have impacted delivery of health services including immunization. Or the delivery of immunization services being impacted by topography of the study areas or by seasonal factors such as monsoons or landslides |
| c. Migration | Discussion of any migratory movement in the study population that may impact access to or uptake of immunization services of children whose caregivers migrate frequently. |
| d. Any existing government or NGO schemes in the study context to support immunization uptake | Discussion of any govt or NGO schemes or programs that encourage uptake of health services for children including immunization. Examples: Govt messaging or sensitization campaigns, etc. |
| e. Baseline immunization rates in the study context | Select this code if the paper states the baseline immunization rates for a given study context. |
| f. Misinformation | Select this code if caregivers or community members mention seeing or hearing bad information about vaccination. Please note, that any information in this code is likely to be captured under social processes codes as well so this information is likely to be double coded |
| g. Socio-economic characteristics | Select this code if the vaccination rates differ by socio-economic characteristics such as income, education, employment status, etc. |
| 6. Program or intervention characteristics |  |
| a. Theory of change | Use this code to select text that describes the theory of change (TOC) of the intervention, if given |
| c. Program activities | Use this code for listing all the program activities/ intervention design |
| d. Program activities specifically targeting any barriers to or drivers of vaccination uptake | Use this code if the paper specifically states that a program or one of its components is targeted towards addressing any specific barrier to immunization. |
| 7. Reasons for intervention failure or success |  |
| Reason for intervention failure | Use these codes for specific statements made by the authors in the conclusion or discussion sections of the paper on why an intervention failed. These statements should complement the findings of the main impact evaluation. For example, a study reporting significant negative or null findings should have included a statement on plausible reasons for intervention failure. |
| a. Not accounting for existing contextual or uncontrollable trends |  |
| b. Implementation failure |  |
| c. Intervention design failure |  |
| d. Study or evaluation design failure |  |
| e. Other reasons |  |
| Reason for intervention success | Use these codes for specific statements made by the authors in the conclusion or discussion sections of the paper on why an intervention was successful. These statements should complement the findings of the main impact evaluation. For example, a study reporting significant positive findings should have included a positive statement on plausible reasons for intervention success. |
| a. Favorable contextual factors |  |
| b. Implementation improvements or success |  |
| c. Intervention design features |  |
| d. Other reasons |  |
| 8. Impacts (on drivers of vaccination, if captured in the study) |  |
| Heterogenous impacts and their reason | Where the intervention only showed an impact among some geographies or sub-populations, sub-sample. Some papers may report overall significant positive, negative or null findings but there may be some parts or populations in the study that reported different results. Use this code if such results are reported |
| Intervention failed to improve barriers to vaccination |  |
| Intervention reduced barriers to vaccination |  |
| Generalizability |  |
| Generalizability_Lack of | Select this code if the study explicitly states that the results cannot be generalized to a larger group of people. |
| Generalizability_present | Select this code if the results of the study are applicable/generalizable to a broader/larger group of people. |
| Sources of information | *If needed, double code the sources* |
| Authors’ observations or Others | If needed, double code the sources |
| Qualitative data | Qualitative data from (caregivers, health providers or other stakeholders – include the quote(s) to know the source of information or include the source if available |
| Quantitative surveys | Quantitative surveys done as part of the study or quantitative datasets used for analysis by the study |
| Secondary literature | which the study cites as other research related to the subject area – include the citation or citation number |

# **Appendix 3: Details of context, and intervention and its impact**

|  | Author | Title &  year of intervention | Contextual information | Applicable intervention codes | Barriers to uptake of vaccination addressed by the intervention | Impacts on drivers and vaccination rates | Generalisability |
| --- | --- | --- | --- | --- | --- | --- | --- |
| 1 | Adamu et al., 2019a | Quality improvement collaborative in primary health care **-** 2019 | **Nigeria:** | BA1. Formal health worker training & education; BB1. Formal health worker involvement in planning & monitoring | Addresses missed opportunities for immunisation | **Drivers:**  Intervention was helpful in addressing missed opportunities for vaccination  **Vaccination**: Not measured | - |
| 2 | Admassie et al., 2009 | Community-based Health Services Extension Programme – 2003 | Ethiopia; Rural | BF1. Health system strategic planning; BA2. Community health worker training and education; BB7. Home visits; BE1. Building & upgrading health clinics | Healthcare experiences | **Vaccination**: BCG, DPT1-3, OPV1-3, Measles, full immunization improved. The effects are higher in women with primary education | - |
| 3 | Aggarwal, 2018 | Prime Minister’s Rural Road Program - 2000 | **India:** Multiple states  -Low income, rural regions | EB1. Non-health or education infrastructure (e.g., electrification) | Construction of roads would lead to increased accessibility to health care facilities | **Drivers:**  -Intervention reduced barriers to vaccination by improving accessibility to health services  **Vaccination**:  -BCG, DPT, measles coverage improved  -No effect on OPV | Considered the data from DLHS- Survey took place in 34 states and union territories in India (excluding Nagaland) |
| 4 | Alatas et al., 2019 | Nationwide immunisation campaign on Twitter **-** 2015 | **Indonesia:** | AA3. Public information campaigns | Knowledge and attitudes about immunisation | **Drivers**:  Increased knowledge and awareness about immunisation  **Vaccination:** Not measured | - |
| 5 | Alhassan et al., 2019 | Bottom-up community engagement intervention – 2013 | Greater Accra and Western regions of Ghana | DA1. Collaborating with whole community; BF1. Health system strategic planning |  | **Vaccination**: Improved child immunization | - |
| 6 | Andersson et al., 2009 | Evidence-based discussion – 2006 | Lasbela, Baluchistan, Pakistan; poor | DA2. Collaborating with selected community groups and networks;  AA2. Short-term sensitization and education campaigns | Think and feel | **Drivers:** Improved knowledge and attitude about vaccination and other intermediate outcomes. But, self-sufficiency/agency of women on vaccination decision, and inclusion of mother in vaccination decision saw no impact.  **Vaccination:** Measles and DPT3 improved, no changes in OPV | - |
| 7 | Andrade et al., 2012 | The Bolsa Família Program: A CCT program **-** 2003 | **Brazil:** | AB1. Material or monetary incentives for caregivers | Parental motivation (poverty alleviation) | **Vaccination**: Not measured | Data were generated from a survey conducted by the Regional Development and Planning Center |
| 8 | Andreoni et al., 2016 | Time preferences to customise incentives for polio vaccinators **-** 2014 | **Pakistan:** | BD1. Material or monetary incentives for health workers | Health worker motivation | **Drivers:**  Formal health worker motivation & capacity improved.  **Vaccination**: Not measured | - |
| 9 | Anjum et al., 2004 | Health education – 1998 | Sikanderabad, Pakistan; low literacy rates | AA2. Short-term sensitization and education campaigns | Think and feel | **Drivers:** Improved knowledge of vaccination. **Vaccination:** Improved BCG and Measles | - |
| 10 | Aquino et al., 2009 | Family health program – 1994 | Brazil | BF1. Health system strategic planning | Provision of comprehensive care through multi-professional teams (physicians, nurses, community health agents, and oral health professionals). They promote, prevent and provide health care to mothers and children (including immunization) | **Vaccination**: OPV 3, DPT3, Measles coverage improved | Generalizability limited |
| 11 | Arifeen et al., 2009 | Integrated Management of Childhood Illness strategy – 2002 | Bangladesh, rural | BA1. Formal health worker training and education; BA2. Community health worker training and education; BF1. Health system strategic planning; AA1. Sustained sensitization and education campaigns; CA1. Faith-based outreach/outreach using local leaders; DA2. Collaborating with selected community groups and networks | Experiences of health services | **Vaccination**: Insignificant result for measles | - |
| 12 | Assegaai et al., 2018 | Ward-based outreach teams – 2011 | North West Province, South Africa | BF1. Health system strategic planning; BA2. Community health worker training and education |  | **Vaccination**: Full immunization improved but not statistically significant, but measles improved | - |
| 13 | Atnafu et al., 2017 | SMS-based exchange application for HEWs **-** 2012 | **Ethiopia** | BG1. New HMIS or dashboard systems (incl. improved data collection);  BG2. Capacity building for existing systems | - | **Vaccination**:  Failed to improve full immunisation coverage | - |
| 14 | Attanasio et al., 2005 | Programme Familias en Acción (FA), a CCT (nutritional subsidy) – 2001-02 | Columbia,  Low socio-economic status | AB1. Material/ monetary incentives for caregivers | Motivation by providing cash transfers to mothers, if they comply with set conditions | **Vaccination**: DPT coverage improved | - |
| 15 | Balasubramaniam et al., 2018 | Systematic screening tool for family planning services among postpartum women attending village health & nutrition days - 2011 | **India:** Jharkhand  -Low income | BA2. Community health worker training and education;  BF1. Health system strategic planning | - | **Vaccination**:  No effect on DPT immunisation | - |
| 16 | Banerjee et al., 2010 | Immunisation campaigns with and without incentives - 2005 | **India:** Rajasthan  -Low income, rural regions | AB1. Material or monetary incentives for caregivers;  BB5. Outreach to vulnerable populations (hard-to-reach, socio-economic status, caste, etc.) | -Establishing regular availability of immunisation services (staff and outreach services). | **Vaccination**:  BCG, **full immunisation,** OPV0, 1, 3, DPT1, 3, Measles coverage improved.  Reduction in unvaccinated children (12-23 months) | - |
| 17 | Banerjee et al., 2020 | Interventions aiming to increase demand for immunisation: incentives, targeted reminders and the leverage of social networks - 2016 | **India:** Haryana  -Low income | AB1. Material or monetary incentives for caregivers;  AB4. Written or pictorial messages (SMS, stickers, flyers etc.) to caregivers;  DA2. Collaborating with selected community groups and networks  (These are single interventions from different arms of the study) | -To address the issues of misunderstanding and decreasing salience community members were selected to spread the information to people  -SMS and voice messages were send aiming to correct misconceptions about immunisation  -Small incentives can be effective in offsetting small costs, thereby ‘nudging’ a mother who may not have strong views about immunising her child | **Drivers:**  -Intervention reduced barriers to vaccination i.e., likelihood of having an information health worker (ASHA), auxiliary nurse midwife, and health guide in the village increased as well as their presence in the facility  - Likelihood of having a health camp organised in village increased  **Vaccination**:  Incentives, Non-incentivised social network experiment-Gossip seeds, independently, have a positive significant effect on **full immunisation**, Measles, DPT1, 2, and 3 coverage (children under 1 yr)  Reminders improved **full immunisatio**n and measles, while other outcomes had statistically insignificant result | Intervention was integrated into the government’s existing routine immunisation services and was tested at a large scale hence, authors suggested that it can be generalised to Haryana and India |
| 18 | Banerjee & Sachdev, 2015 | Prime Minister’s Rural Road Program (or Pradhan Mantri Gram Sadak Yojana)  -2000 | **India:** Multiple states  -Low income, rural region | EB1. Non-health or education infrastructure (e.g., electrification) | Construction of roads could improve health care supply, increase household income, increase awareness, and improve social interaction in the village, all of these in turn increase usage of preventive health care | **Drivers:**  -Intervention reduced barriers to vaccination by improving availability of frontline health workers (ASHA and ANMs)  **Vaccination**: Not measured | Considered the data from DLHS- Survey took place in 34 states and union territories in India (excluding Nagaland) |
| 19 | Bangure et al., 2015 | SMS reminders – 2013 | Kadoma city, Zimbabwe | AB4. Written or pictorial messages (SMS, stickers, flyers etc.) to caregivers |  | **Vaccination**: Intervention improved immunization and timeliness |  |
| 20 | Banwat et al., 2015 | Peer education **-** 2014 | **Nigeria:** | AA1. Sustained sensitisation & education campaigns;  BB7. Home visits;  DB1. Community tracking & registering |  | **Drivers:**  Improvement in knowledge about routine vaccination  **Vaccination**:  Full immunisation coverage improved but it was not statistically significant | - |
| 21 | Barham et al., 2007 | 1. Progresa (A Oportunidades - formerly Progresa (A CCT)- 1997   CCT) – 1997 | Mexico, rural poor | AB1. Material/ monetary incentives for caregivers | Motivation by providing cash transfers to households. | **Vaccination**:  -Timely BCG and Measles improved after 6 months of intervention implementation but, after 12 months the results were insignificant.  -BCG coverage was insignificant.  -Measles coverage at 12 months improved significantly in households with less educated mothers. -Measles at 6 & 12 months improved in households living away from 5.5 KM. | - |
|  |  | Red de Protección Social––RPS (A CCT) – 2000 | Nicaragua; Rural poor | AB1. Material/ monetary incentives for caregivers | Motivation by providing cash transfers to households. | **Vaccination**:  -On time vaccination of OPV3, full vaccination improved after 5 months of intervention implementation.  -At 12 months, OPV3 showed statistically significant positive impact. -At 24 months OPV3 and full immunization improved but results was not significant.  -OPV3, DPT3, Measles and full immunization coverage improved significantly among those who lived far from health centre and with less educated mothers | - |
| 22 | Basinga et al., 2011 | Pay-for-performance **-** 2006 | **Rwanda:** | BD5. Pay-for-performance scheme | Improve quality of health services | **Vaccination**:  Failed to improve full immunisation coverage | The Rwanda Demographic and Health Surveys, a large, nationally representative household survey |
| 23 | Beck, Pulkki-Brännström & Sebastián, 2015 | 1. Non-contributory, universal and unconditional cash transfer - 2011 | **India:** Madhya Pradesh  -Low income | AB1. Material or monetary incentives for caregivers | -Cash transfers may have an effect on health resilience, enabling people to have greater resistance to sickness, through better diets, more regular medical treatment, improved sanitation, & diminished stress due to economic insecurity. | **Vaccination**:  Failed to improve full immunisation coverage in children (6 months and 5 years) | - |
| 24 | Berhane & Pickering, 1993 | Reminder stickers – 1991 | Lideta & Nefas-Silk districts, Addis Ababa, Ethiopia; urban | AB4. Written or pictorial messages (SMS, stickers, flyers etc.) to caregivers |  | **Vaccination**: Improved DPT1 to 3 dropout rates | Limited to study districts |
| 25 | Bhuiya et al., 2016 | Self-help for health: to work with existing rural self-help organisations - 1994 | **Bangladesh:**  -Low income,  Low education,  Climatic events- cyclones, tidal waves & floods,  study site belonged to the most religiously conservative areas | AA1. Sustained sensitisation & education campaigns;  BA1. Formal health worker training & education;  BA2. Community health worker training & education;  BE1. Building & upgrading health clinics;  BG2. Capacity building (e.g., training) for existing systems;  DA1. Collaborating with whole community;  DA2. Collaborating with selected community groups & networks | -raising awareness related to health promotion, prevention  -health care seeking and enhancing the capacity of village clubs, youth clubs, profession-based associations and cooperatives, and mosque committees | **Drivers**  -Self-help groups collective action in supporting immunisation services improved  - Improvement in mobilisation of people by self-help groups for immunisation  - improvement in possessing immunisation card  **Vaccination**  -Full immunisation coverage increased | As the study was conducted in limited number of villages in rural Bangladesh, the findings cannot be generalised to other regions of the country, however, the intervention can be adopted in low income-settings |
| 26 | Bhuiya et al., 2006 | Maternal & Child Health (MCH) Handbook – 2002 | Bangladesh | AB4. Written or pictorial messages (SMS, stickers, flyers etc.) to caregivers | Handbook serves the purpose of information provision, record keeping and referral | **Drivers**: Improvement in mother’s knowledge and attitude  **Vaccination:** Child vaccination improved | - |
| 27 | Biemba et al., 2016 | Community health assistants (or CHWs) programme – 2010 | Luwingu and Senanga districts of Zambia; rural hard-to-reach areas | BF1. Health system strategic planning; BA2. Community health worker training and education |  | **Vaccination**: Immunization improved | - |
| 28 | Binyaruka et al., 2015 | Pay-for-performance – 2012 | Pwani, Tanzania | BD5. Pay-for-performance schemes | Improved health staff motivation and resources, thereby changed health staff behavior & subsequently improved healthcare quality and reduce cost of services | **Vaccination**: No effect on immunization (OPV0, measles, DPT3) and people’s experiences of care | - |
| 29 | Björkman & Svensson, 2009 | Community-based monitoring – 2005 | Uganda; rural | DA1. Collaborating with whole community; BF1. Health system strategic planning | To increase the efforts by health staff | **Vaccination**: Improved immunization and intermediate outcomes | - |
| 30 | Bolam et al., 1998 | Postnatal health education for mothers – 1994 | Nepal | AA2. Short-term sensitization and education campaigns; BA2. Community health worker training and education | Knowledge and practices of immunization would improve | **Vaccination**: Not statistically significant change in immunization | - |
| 31 | Bonfrer et al., 2014 | Performance based ﬁnancing – 2006 | Burundi; post-conflict, poor | BD5. Pay-for-performance schemes | Incentives motivates providers to put more efforts in specific activities and increases the resources thereby improving MCH services | **Vaccination**: Full immunization, BCG, OPV, DPT, Measles improved | - |
| 32 | Borkum et al., 2014 | Team-Based Goals and Performance Based Incentives (TBGI) (part of Ananya Program) - 2012 | **India:** Bihar  -Low income | BD1. Material or monetary incentives for health workers; BD2. Non-material incentives for health workers | -TBGI to increase motivation, teamwork,  interactions, and joint problem-solving among health workers, to lead subsequently to more and better services to households and eventually to improvements in maternal and child health outcomes  -Health workers to visiting more marginalised communities to provide more equitable services  -Improved interactions with FLWs could affect  maternal and child health-related behaviours is by increasing beneficiaries’ knowledge of issues related to their health and the health of their child, and of desirable health behaviours | **Drivers:**  -Increased attendance at sub-centres and increased FHW interaction with the communities.  -Intervention failed to improve barriers to vaccination- i.e., female health workers discussing about immunisation with the households  **Vaccination**:  No effect on DPT3 and full vaccination | As the study was conducted in a single district, i.e., Begusarai, authors suggested that it may not be generalised to other parts of Bihar. |
| 33 | Bradley & Igras, 2005 | Client-Oriented, Provider Efficient Services (A quality improvement approach) – | Guinea and Kenya | BF1. Health system strategic planning; BA1. Formal health worker training and education. | Giving power of change to health staff, building capacity and facilitating them with actions, health staff would take actions to improve services. | **Drivers:** Improved quality indicators such as staff capacity building, staff satisfaction and performance, outreach services, upgrading infrastructure, record keeping, discussing immunization schedule to reduce missed vaccination | - |
| 34 | Brown et al., 2016 | Community Health Nurse-Led Intervention - 2012 | **Nigeria:**  -Low income, Urban | AB3. Automated voice messages to caregivers;  BA1. Formal health worker training & education | - | **Vaccination**:  Cell phone reminder/recall; Combination of cell phone reminder/recall and PHC immunization providers’ training improved full immunisation coverage.  PHC immunization providers’ training alone failed to improve full immunisation coverage | Study findings can only be generalised to urban and suburban population in the city of Ibadan, Nigeria |
| 35 | Brown et al., 2017 | Training immunization providers - 2015 | **Nigeria:** | BA1. Formal health worker training and education | - | **Drivers:**  Improvement in knowledge and self-reported practice  **Vaccination**: Not measured | - |
| 36 | Brugha & Kevany, 1996 | Home visits – 1991 | Nkawkaw, Kwahu Praso, & Akwasiho towns of Ghana | BB7. Home visits; AA2. Short-term sensitization and education campaigns | Improves the contact of health workers and caregivers, improves record keeping, help in tracking children and motivating caregivers thereby increasing service uptake. | **Vaccination**: Improved immunization rates | - |
| 37 | Busso et al., 2015 | The Programa de Extensión de Cobertura: Coverage extension programme **-** 2011 | **Guatemala:**  -Low income, rural | BB7. Home visits;  BG1. New HMIS or Dashboard systems (Inc. improved data collection) | Parental reminders in informing the community about the outreach services | **Vaccination**:  Full immunisation coverage improved | - |
| 38 | Cahyadi et al., 2018 | Program Keluarga Harapan (PKH or “Hopeful Family Program”: A conditional cash transfer (CCT) program - 2007 | **Indonesia**:  -Low income | AB1. Material or monetary incentives for caregivers; | Parental motivation (poverty alleviation) | **Vaccination**  -Full immunisation improved at 6-year follow-up but no effect on 2-year follow-up | - |
| 39 | Carnell et al., 2014 | Essential Services for Health in Ethiopia Project through ‘Three Pillars’ Approach i.e., (i) strengthen health systems, (ii) improve health workers’ performance, and (iii) engage the community **-** 2004 | **Ethiopia:**  -Low income | BA1. Formal health worker training & education;  BA2. Community health worker training & education;  BD2. Non-material incentives for health workers;  BF1. Health system strategic planning;  BG1. New HMIS or Dashboard systems (Inc. improved data collection) | - | **Drivers:**  Information about immunisation heard by the caregivers increased; attitudes about immunisation increased;  Presence of health posts increased  **Vaccination**:  DPT3 and Measles coverage improved | - |
| 40 | Carvalho et al., 2014 | Janani Suraksha Yojana: A Conditional Cash Transfer Program - 2005 | **India:** Multiple states | AB1. Material or monetary incentives for caregivers  BD1. Material or monetary incentives for health workers | -Increased interaction with the health system through institutional deliveries could indirectly lead to an increase in childhood immunizations | **Vaccination**:  BCG, OPV0, 1, 3, DPT1, 3, Measles, **full immunisation** improved.  Reduced unvaccinated children. | Considered the data from DLHS- Survey took place in 34 states and union territories in India (excluding Nagaland) |
| 41 | Chandir et al., 2010 | Food coupon incentives **-** 2006 | **Pakistan:**  -Low income, Low literacy | AB1. Material or monetary incentives for caregivers | Parental motivation (poverty alleviation) | **Vaccination**:  DPT 3 timely completion improved | Incentive-based strategy may yield desired results in geographically targeted areas with high poverty and thus results can be generalised to low socio-economic populations in LMICs with low immunisation coverage |
| 42 | Chansa et al., 2015 | Results-based financing – 2008 | Katete District, Zambia | BD5. Pay-for-performance schemes | Motivation to improve performance of health staff | **Drivers:** Improved some of the intermediate outcomes pertaining to healthcare quality and health system strengthening  **Vaccination**: Improved immunization outcomes | - |
| 43 | Chen, Chindarkar & Xiao, 2019 | Jyotigram Yojana (JGY): A rural electrification program - 2003 | **India:** Gujarat | EB1. Non-health or education infrastructure (e.g., electrification) | -receiving health information (via television and internet) & utilisation of health services and -a supply-side prerequisite for health facilities to provide safe and good quality health services e.g., availability of cold storage facilities.  -“time endowment” effect on households: electrification makes households more efficient in labour-intensive activities, free time then can potentially be allocated to accessing health services | **Drivers:**  -Intervention reduced barriers to vaccination i.e., PHC reporting availability of electricity improved, thus need for generator decreased and functioning of cold chain improved; this improvement of health facilities led to improved health services utilisation  **Vaccination**:  -DPT1, OPV1, BCG, Measles, OPV3 coverage improved  **-**No effect on DPT3. | Considered the data from DLHS- but only to Gujarat |
| 44 | Chen et al., 2016 | A Smartphone app (EPI app) – 2013 | rural Sichuan Province, China | BG1. New HMIS/Dashboard systems (incl. improved data collection); AB3. Automated voice messages to caregivers; AB4. Written or pictorial messages (SMS, stickers, flyers etc.) to caregivers | Motivation and reminders to caregivers and tracking missed vaccination by health system | **Vaccination**: Full immunization, BCG, DPT3, OPV3, Measles were not significant | Lack of generalizability |
| 45 | Costa-Font & Parmar, 2017 | Village health and sanitation committees (VHSCs): A political agency - 2005 | **India:** Multiple states | DA1. Collaborating with whole community | -VHSCs develop village health plans and manage an untied fund to enable local planning and action. They organise health-promotion activities and mobilise pregnant women and children to access maternal and health care services | **Vaccination**:  Improved BCG coverage, but not OPV, DPT coverage | Considered the data from DLHS- Survey took place in 34 states and union territories in India (excluding Nagaland) |
| 46 | Cristia et al., 2011 | The Programa de Extensión de Cobertura: Coverage extension programme – Contracting out Primary Care Services **-** 1996 | **Guatemala:**  -Low income, underserved rural areas, indigenous population | BB5. Outreach to vulnerable populations (hard-to-reach, SES, caste etc);  BF1. Health system strategic planning;  DA1. Collaborating with whole community | Address the transport barrier by providing the services in the community and information gaps | **Vaccination**:  BCG, DPT1, OPV1, Measles coverage improved | Data from Guatemalan Living Standards Measurement Surveys |
| 47 | Cristia, Prado, Peluffo, 2015 | The Programa de Extensión de Cobertura: Coverage extension programme -Contracting in and Contracting out Basic Health Services **-** 1996 | **Guatemala:**  -Low income, rural, and primarily indigenous communities | BB5. Outreach to vulnerable populations (hard-to-reach, SES, caste etc);  BF1. Health system strategic planning;  DA1. Collaborating with whole community | Address the transport barrier by providing the services in the community and addressed inadequate quality of services by supervising the services | **Vaccination**:  Contracting-in and contracting-out improved BCG, DPT1 and OPV1.  Contracting-in improved DPT3 and OPV3, however, contracting-out did not produce statistically significant result. | National Surveys of Maternal and Child Health |
| 48 | Cristia, Evans, Kim, 2015 | The Programa de Extensión de Cobertura: Coverage extension programme – Contracting out Mobile Medical Teams **-** 1996 | **Guatemala:**  -Low income, underserved mostly rural, poor and indigenous | BB5. Outreach to vulnerable populations (hard-to-reach, SES, caste etc);  BF1. Health system strategic planning;  DA2. Collaborating with selected community groups | Address the transport barrier by providing the services in the community | **Vaccination**:  BCG, DPT1, OPV1, Measles coverage improved | Data from Guatemalan Living Standards Measurement Surveys (LSMS) data |
| 49 | De Walque et al., 2017 | Performance based ﬁnancing – 2012 | Cameroon | BD5. Pay-for-performance schemes |  | **Drivers:** some of the intermediate outcomes related to quality of services, availability of vaccines improved  **Vaccination**: Immunization outcomes improved | - |
| 50 | Dicko et al., 2011 | Intermittent preventive treatment of malaria – 2006 | Kolokani, Mali | BF1. Health system strategic planning | Caregivers are concerned about malaria and thus, antimalaria treatment has better acceptability | **Vaccination**: DTP3, Measles full vaccination coverage increased. BCG increased but insignificant | - |
| 51 | Dissieka et al., 2019 | Mobile phone message reminders – 2014 | Korhogo district, Côte d’Ivoire; majority illiterate | BG1. New HMIS/Dashboard systems (incl. improved data collection); AB3. Automated voice messages to caregivers; AB4. Written or pictorial messages (SMS, stickers, flyers etc.) to caregivers. |  | **Vaccination**: DPT1-3, Measles, full immunization improved | Lack of due to areas with good network was selected |
| 52 | Djibuti et al., 2009 | Supportive supervision of immunization managers -  2005 | Geogia | BA1. Formal health worker training and education;  BF1. Health system strategic planning | Providing supportive supervision (guidelines, training, monitoring performance, & funding for travel and communication) to improve the competency of immunization managers | **Drivers:** Perceived knowledge about supportive supervision significantly improved among immunization managers. Perceived barriers to implement supportive supervision (e.g., availability of resources, availability of format) improved. Decrease in DPT vaccine wastage.  Qualitative findings suggest improved management approaches, clarification of roles and responsibilities and others. Whereas, reaching remote areas because of transport difficulties and low technical capacity of local health providers were reported.  **Vaccination**: DPT3 coverage improved | - |
| 53 | Demilew et al., 2021 | Supportive Feedback and Nonmonetary Incentives: “Protected Children” posters to track immunisation achievement and drop-outs **-** 2016 | **Ethiopia:**  -Civil unrest, rural | AB2. Non-monetary incentives for caregivers;  BB1. Formal health worker involvement in planning & monitoring;  BB3. Paper-based tracking;  BD4. Written or pictorial messages for health workers | -Positive reinforcement for health extension workers (HEWs) leading to improvements in self-efficacy and increased engaged with the community;  -Poster helps HEWs track the children who missed timely vaccination;  -Poster is visible to the community, which makes HEWs act and thus increase home visits made by them to motivate parents in immunising children;  -Behaviourally-informed feedback and non-monetary rewards for caregivers | **Drivers:**  Increase in HEW visits  **Vaccination**:  No statistically significant treatment effect on DPT3, incomplete vaccination, full immunisation coverage | Oromaria had low immunisation rates (12%) hence findings may be generalisable to low performing regions of Ethiopia |
| 54 | Dipeolu, 2017 | SMS reminders **-**2014 | **Nigeria:** | AB4. Written or pictorial messages (SMS, stickers, flyers, etc.) to caregivers | Parental motivation | **Drivers:**  Improvement in knowledge and attitude about vaccination but not statistically significant  **Vaccination**:  Full immunisation coverage improved.  Failed to improve timely completion of immunisation | Findings can be generalised |
| 55 | Domek et al., 2019 | SMS-based vaccination reminder system - 2016 | **Guatemala:**  -Low income, Political instability | AB4. Written or pictorial messages (SMS, stickers, flyers, etc.) to caregivers | Parental reminders | **Vaccination**:  Visit completion for immunisation did not improve.  Timely vaccination visits improved. | - |
| 56 | Drain et al., 2006 | Introduction of auto-disabled syringes in national immunization programme – 2000 | Antananarivo & Fianarantsoa, Madagascar | BF2. Vaccination guidelines;  BA1. Formal health worker training and education. | Increase efficiency of health workers especially in outreach sessions | **Vaccination**: Increased vaccination coverage rates by increasing number of vaccines administered on non-routine vaccination days | - |
| 57 | D'Souza & Umarani, 2014 | Teaching package on vaccine preventable diseases - 2012 | **India:** Karnataka | AA2. Short-term sensitization and education campaigns | -Education about vaccine preventable diseases | **Drivers:**  -Intervention reduced barriers to vaccination i.e., improved mother’s knowledge  **Vaccination**: Not measured | No mention  (Small sample size & participants purposively selected from hospitals) |
| 58 | Eichler et al., 2007 | Performance-Based Incentives for Health – 1995 | Haiti | BD5. Pay-for-performance schemes | Health services might improvise the services, motivates the health workers, reach to underserved people and use resources. | **Vaccination**: Full immunization coverage improved | - |
| 59 | Ekhaguere et al., 2019 | SMS and voice calls **-** 2016 | **Nigeria:** | AB3. Automated voice messages to caregivers;  AB4. Written or pictorial messages (SMS, stickers, flyers, etc.) to caregivers | - | **Vaccination**:  Failed to improve DPT3 coverage. Improved full immunisation coverage, timeliness for DPT3 and Measles | Study restricts the findings to women owing mobile phone |
| 60 | Engineer et al., 2016 | Pay-for-performance – 2010 | Afghanistan | BD5. Pay-for-performance schemes | Intended to improve health worker motivation and satisfaction thereby improve user’s satisfaction | **Vaccination**: No improvement in DPT3 | - |
| 61 | Eze & Adeleye, 2015 | SMS reminders **-** 2010 | **Nigeria:** | AB4. Written or pictorial messages (SMS, stickers, flyers, etc.) to caregivers | - | **Vaccination**:  Improved timeliness and coverage of DPT3 | - |
| 62 | Findley et al., 2013a | IMCI through Community volunteers and CHWs **-** 2009 | **Nigeria:** | AA1. Sustained sensitization and education campaigns;  AA3. Public information campaign;  BA1. Formal health worker training & education;  BA2. Community health worker training & education;  BF1. Health system strategic planning;  DA1. Collaborating with whole community | - | **Drivers:**  Community relying on CHWs for health services improved;  Availability of midwives improved  **Vaccination**:  Improved newborn vaccination coverage | - |
| 63 | Goel et al., 2011 | Muskaan Ek Abhiyan (The Smile Campaign) - 2007 | **India:** Bihar | AA1. Sustained sensitization and education campaigns;  AA3. Public information campaigns,  BA1. Formal health worker training and education,  BA2. Community health worker training and education,  BB5. Outreach to vulnerable population (hard-to-reach, SES, cast etc.),  BB7. Home visits,  BD1. Material or monetary incentives for health workers,  BF1. Health system strategic planning | -Improvement in immunisation service provision e.g., increase in number of days providing immunisation and outreach activities  -Logistic distribution plan  -Mobilisation activities  -Tracking of children  -Awareness programs through women’s groups  -Intensiﬁed communication activity and media attention  -Performance based incentive to service providers for motivation  -Enhanced political commitment and budgetary support  -Training and improvement in supply of health workers  -Strengthening monitoring and supervision of health staff | **Drivers:**  -Intervention reduced barriers to vaccination i.e.,  -supply of health workers such as Anganwadi workers and ASHAs,  -improved functioning of cold chain  -having a vaccinator present at the vaccination site improved  -Vaccine availability at the vaccination centre improved  -Anganwadi workers were observed mobilising beneﬁciaries  -improvement in beneficiary tracking  **Vaccination**:  **Full immunisation,** BCG, DPT3, OPV3, Measles coverage improved among 12-23 months old | Intervention is feasible and can be successfully implemented in underserved areas of India |
| 64 | Goodson et al., 2012 | Integrating insecticide-treated bed nets in a vaccination campaign – 2007 | Madagascar; majority rural | BC1. National/sub-national immunisation days; AB1. Material/ monetary incentives for caregivers |  | **Vaccination**: Improved measles coverage, especially among poorest | - |
| 65 | Gurley et al., 2020 | Community-led video education - 2016 | **India:** Uttar Pradesh  -Low income, rural (includes hard-to-reach population) | AA2. Short-term sensitization and education campaigns;  BB5. Outreach to vulnerable populations (hard-to-reach, SES, caste, etc.);  BA2. Community health worker training and education | -Intervention would have effect on mothers’ attitudes and beliefs, mothers’ networks and social norms, and community attitudes and social norms  -intervention package targeted husbands, families (including mothers-in-law) and communities by targeting behavioural determinants of vaccination, including knowledge, attitudes and norms | **Drivers:**  -Intervention reduced barriers to vaccination i.e.,  -intervention improved maternal knowledge of vaccination. Improvement in husband’s knowledge was also found.  -Among HTR population intervention could increase awareness of vaccination  -engagement of ASHA with the community increased (qualitative finding)  -changes in perception of vaccine-induced side-effects were reported (qualitative finding)  -Intervention failed to reduce barriers to vaccination i.e., -among Muslim participants, intervention could not alleviate belief that vaccine cause infertility  -intent to vaccinate was increased but it was not statistically significant and measured separately for mothers and family members (intent to vaccinate was high at baseline)  -ASHA’s knowledge and skills improved but was not statistically significant  **Vaccination**:  -Intervention was successful in improving DPT1-DPT3 dropouts, & DPT3 coverage.  -Intervention failed to improve **fully immunised children** and vaccination timeliness. | Mobilising women to attend the meetings depends on the context, therefore intervention may work in rural-settings with strong community health worker cadres and existing women’s groups but may not work in urban-setting. Secondly, intervention may work in the contexts in which mothers play a larger role in immunisation decision-making. Lastly, intervention can be scaled-up at low cost if implemented in government services |
| 66 | Habib et al., 2017a | Community engagement and integrated health & polio immunisation campaigns - 2014 | **Pakistan:**  -Area with high risk for polio certified by WHO;  Conflict affected region | BA2. Community health worker training & education;  BB5. Outreach to vulnerable populations (bard-to-reach, SES, caste etc.);  CA1. Faith-based outreach or outreach using local leaders; DA2. Collaborating with selected community groups & networks | Improvement in accessibility through outreach activities and gaining trust of community through community engagements | **Vaccination**:  OPV3 and full vaccination coverage improved | The findings can be generalised to conflict affected regions with limited community engagement & the regions that face polio resistance |
| 67 | Hagiwara et al., 2012 | MCH Handbook – 2008 | Jericho and Ramallah Governorates in Palestine, conflict affected | AB4. Written or pictorial messages (SMS, stickers, flyers etc.) to caregivers; BA1. Formal health worker training and education; AA2. Short-term sensitization and education campaigns | Handbook serves the purpose of information provision, record keeping and thereby empowering women in healthcare knowledge and attitude | **Vaccination**: No impact on immunization outcome | - |
| 68 | Hategeka et al., 2019 | RapidSMS: Community health worker m-health monitoring system **-** 2013 | **Rwanda:** | AB4. Written or pictorial messages (SMS, stickers, flyers etc.) to caregivers;  BD4. Written or pictorial messages for health workers; BG1. New HMIS or dashboard systems (incl. improved data collection) | Program intended to improve the quality of care. It addresses three delays: seeking care (decreased response time), reaching care (improved tracking) and ineffective intervention (better decision-making by policymakers) | **Vaccination**:  Failed to improve BCG and OPV0 coverage | - |
| 69 | Hu et al., 2017 | Parental vaccination education – 2014 | Zhejiang province, China | AA2. Short-term sensitization and education campaigns | Think and feel | **Drivers:** improved vaccination knowledge  **Vaccination**: Full vaccination, OPV3, DPT3, measles and timeliness of vaccination improved | - |
| 70 | Huillery & Seban, 2017 | Performance based ﬁnancing – 2010 | Haut-Katanga district, DRC; poor, conflict-affected | BD5. Pay-for-performance schemes | Improves performance of health workers thereby attracting more users and health outcomes | **Drivers:** health worker performance (organized immunization session at the facility, outreach sessions and staff attendance) improved but their job satisfaction and service quality did not  **Vaccination**: no effect on children receiving at least one immunization and BCG scar | - |
| 71 | Hutchison et al., 2006 | Smiling Sun Campaign, a National Health Communication Program – 2001 | Bangladesh | AA3. Public information campaigns; AB4. Written or pictorial messages (SMS, stickers, flyers etc.) to caregivers | Think and feeling | **Vaccination**: DPT3 and Measles improved | - |
| 72 | Janssens, 2011 | Mahila Samakhya, a women’s empowerment program - 1992 | **India:** Bihar  -Low income  -Low education, rural region | AA1. Sustained sensitization and education campaigns;  DA2. Collaborating with selected community groups and networks | -Intervention aimed to set up women’s groups in rural villages in order to educate and empower women, women from socially and economically marginalised backgrounds, such as the Scheduled Castes.  -assist the women in identifying their own needs and solutions e.g., activities such as literacy trainings, setting up savings and credit groups, and informal primary schools for girls  -others people might beneﬁt from improved facilities or increased awareness in the community. -improvement in knowledge on health and hygiene | **Drivers:**  -Intervention reduced barriers to vaccination e.g.,  -village women protested absence of health staff and received assurance that absenteeism will not be repeated, similarly protested against non-functioning of PHCs, non-availability of medicines and doctors at PHCs.  -ANMs have become more regular with their village visits  -Improvement in functioning of the PHC/sub PHCs  -Group members assist in immunisation and other drives to mobilise people  **Vaccination**:  -Improved **DPT,** Measles, BCG coverage  **-**No effect on OPV | - |
| 73 | Johri et al., 2020 | Social and behaviour change communication interventions delivered face-to-face and a mobile phone - 2018 | **India:** Uttar Pradesh  -Low income, rural region | AA2. Short-term sensitization and education campaigns;  AA3. Public information campaigns;  AB3. Automated voice messages to caregivers;  BB7. Home visits;  DA1. Collaborating with whole community | -Tika Vaani (vaccine voice in Hindi) model to educate beneficiaries about immunisation and basic child health themes, dispel misinformation, and empower households to better care for their children and themselves by providing context-appropriate audio messages delivered via automated phone calls and face-to-face meetings separately for men and women  -child vaccination reminders were sent to target group  households.  -volunteers visiting the households to improve knowledge, awareness and attitudes towards immunisation  -Community mobilisation events involving activities, theatre and discussion groups to identify problems related to immunisation in the communities, discuss possible causes and solutions, and give feedback.  -A toll-free number for immunisation enabling anonymous queries and feedback | **Drivers:**  -Intervention reduced barriers to vaccination i.e.,  -General knowledge about vaccination improved  -Knowledge of the vaccination calendar increased  -Intervention failed to improve barriers to vaccination i.e., ability to interpret the child’s immunisation card did not improve  **Vaccination**: Not measured | If the technical delivery and content is adapted as per local needs, the mHealth interventions can achieve reach and improve knowledge even in highly underprivileged populations, however, it depends on women’s access to mobile phones and technical familiarity  - Audio messaging was amenable to culture-specific contextualization and an edutainment approach |
| 74 | Kawakatsu et al., 2015 | MCH handbook – 2011 | Siaya, Ugenya, Gem and Kisumu West Districts, Nyanza Province, Kenya; Rural | AB4. Written or pictorial messages (SMS, stickers, flyers etc.) to caregivers | Provision of health knowledge | **Drivers:** Improved health knowledge and health-seeking behavior.  **Vaccination**: No difference in full vaccination | - |
| 75 | Kazi et al., 2018 | Mobile phone text message reminders **-** 2013 | **Pakistan:**  -Low income | AB3. Automated voice messages to caregivers;  AB4. Written or pictorial messages (SMS, stickers, flyers etc.) to caregivers | Parental motivation | **Vaccination**:  -DPT 1 coverage improved.  -DPT2, DPT3 and timeliness were not statistically significant. | - |
| 76 | Kusuma et al., 2017 | Program Keluarga Harapan (PKH): a CCT **-** 2007 | **Indonesia**:  -Low income | AB1. Material or monetary incentives for caregivers | Parental motivation (poverty alleviation) | **Drivers:**  Provided a larger impact on low educated mothers as compared to higher educated mothers. Larger (4-point increase) impact was observed among urban residents compared to rural residents; this can be explained because of better access to public health services  **Vaccination:** Coverage of full vaccination, BCG, OPV3, DPT3, and Measles improved for children under 12 months of age.  While, only OPV3 and Measles coverage improvement seen among children 12-23 months | Follow-up data were based on a large-scale evaluation (approximately 14,000 households) done by National Planning Agency and World Bank |
| 77 | Lee, 2015 | Incentives for health workers – 2010 | Zambia | BD2. Non-material incentives for health workers | Intervention might affect performance | **Drivers**: Increased some of the intermediate outcomes such as CHWs household visits, CHW skills,  **Vaccination**: Improved OPV, full immunization  BCG and measles improved | - |
|  |  | Incentives for health workers – 2010 | Zambia | BD2. Non-material incentives for health workers | By conferring employer recognition, by enhancing social visibility, and by facilitating social comparison | **Drivers:** No effect on CHW performance | - |
|  |  | Incentives for health workers – 2014 | Muratganj. Kaushambi District, Uttar Pradesh, India | BD2. Non-material incentives for health workers | Intrinsic motivation may affect external motivation. It may increase productivity | **Drivers:** Increase home visits | - |
| 78 | Loevinsohn, 1986 | Food supplements – 1985 | Nicaragua, rural poor | AB1. Material/ monetary incentives for caregivers | Motivation by providing food supplements to households. | **Vaccination:** BCG coverage was insignificant | - |
| 79 | Manyazewal et al., 2018 | Continuous quality improvement (CQI) interventions **-** 2016 | **Ethiopia:** | BA1. Formal health worker training & education;  BF1. Health system strategic planning;  BG2. Capacity building for existing systems | Health workers trained in health services increased; increase in cold chain officers, immunisation experts; healthcare facility having micro plan, standard operating procedures and national immunisation guidelines increased; availability of logistics, supply and equipment increased; electronic database system for immunisation increased; immunisation technical working group and partners’ forum between government and non-government organisation increased; increased in updated EPI monitoring charts, completeness of monthly report, and monitoring and evaluation and data management activities; the national capacity to deliver immunization programme and services was increased at zone, district and healthcare facility (as compared to other regions, Gambella and SNNPR saw better improvements) | **Vaccination**:  DPT3, Measles, OPV, and BCG improved.  Full immunisation produced insignificant result. | The study was restricted to six out of nine states hence, findings cannot be generalisable |
| 80 | Mathanga et al., 2009 | Integrating insecticide-treated bed nets in routine vaccination – 2006 | Mwanza and Phalombe in Malawi; Rural | AB1. Material/ monetary incentives for caregivers | Incentivizing caregivers | **Vaccination**: Improved timeliness and full immunization |  |
| 81 | Mayumana et al., 2017 | Pay-for-performance – 2011 | Pwani, Tanzania | BD5. Pay-for-performance schemes | improve provider motivation and supervision | **Drivers:** Qualitative data revealed that managers were responsive for vaccine stockouts but no impact on outreach services. Quantitative data suggested vaccine stockouts reduced but no impact on facilities having outreach services | - |
| 82 | Mazumder et al., 2014 | Integrated Management of Neonatal and Childhood Illness programme - 2003 | **India:** Haryana  -Low education | AA1. Sustained sensitization and education campaigns;  BA1. Formal health worker training and education;  BA2. Community health worker training and education;  BB7. Home visits;  BC1. National or sub-national immunisation days;  BD5. Pay-for-performance schemes;  BF1. Health system strategic planning | -Health worker training for improvement in skill  -Strengthening the health system: supervision of CHWs and nurses, vacant supervisor posts were filled, supervisors were trained  -task based incentives to health workers  -Home visits by CHWs  -ASHA’s ran women’s group meetings to raise awareness about newborn care practices | **Drivers:**  -Intervention reduced barriers to vaccination i.e., knowledge and skill improvement among ANMs and Anganwadi workers  **Vaccination:**  No effect on BCG, DPT3, Measles coverage | - |
| 83 | Memon et al., 2015 | Community-based perinatal and newborn preventive care package (community education & awareness creation) implemented through lady/ community health workers **-** 2003 | **Pakistan:**  -Conflict affected, Low income, Climatic events | AA1. Sustained sensitisation & education campaigns;  BA2. Community health worker training & education;  DA2. Collaborating with selected community groups & networks | Parental awareness | **Vaccination**:  Full immunisation coverage improved | - |
| 84 | Modi et al., 2019 | mHealth intervention “ImTeCHO” - 2015 | **India:** Gujarat  -Tribal rural region | AB4. Written or pictorial messages (SMS, stickers, flyers etc.) to caregivers;  BD3. Automated voice messages to health workers;  BD4. Written or pictorial messages (SMS, stickers, flyers etc.) to health workers;  BG1. New HMIS or Dashboard systems (incl. improved data collection);  BG2. Capacity building (e.g., training) for existing systems | -registers children  -ASHA’s receive upcoming immunisation reminders  -ASHA’s receive daily work schedule  -ASHA’s show educational video clips to family during home visits  -decision support system to ASHA’s  -Track immunisation  -ensured timely payment to ASHA’s | **Vaccination**:  No effect on DPT 3 coverage | - |
| 85 | Mohan et al., 2011 | Integrated Management of Neonatal and Childhood Illness programme - 2005 | **India:** Multiple states | BA1. Formal health worker training and education;  BA2. Community health worker training and education;  BB7. Home visits | -training for building of individual skills  -provision of care by trained providers | **Vaccination**:  No effect on full immunisation coverage | Considered the data from DLHS- Survey took place in 34 states and union territories in India (excluding Nagaland) |
| 86 | Mohanan et al., 2020 | Social accountability interventions - 2016 | **India:** Uttar Pradesh | BB7. Home visits;  DA1. Collaborating with whole community | -provide information to community members on services they are entitled to receive  -facilitate citizen engagement with service providers and local officials through community meetings  -both these activities in turn improve service delivery | **Drivers:**  -Intervention reduced barriers to vaccination i.e., satisfaction with healthcare providers was improved but it was not statistically significant  **Vaccination**:  -Full immunisation (12-23 months), DPT3, improved.  No effect on Measles.  (Same results in both arms)  **-**OPV3 coverage improved in information + facilitation arm but not information only arm. | - |
| 87 | More et al., 2012 | Community mobilisation - 2006 | **India:** Maharashtra  -Low income, urban slums | DA1. Collaborating with whole community | -identify problem, potential solutions, planning, implementing and monitoring potential solutions and sharing information with others | **Vaccination**:  Statistically insignificant result for BCG coverage | - |
| 88 | More et al., 2017 | Local resource centres delivering integrated activities - 2012 | **India:** Maharashtra  -Low income, urban slums | BB7. Home visits;  DA1. Collaborating with whole community | -a series of participatory learning and action and resource-mapping exercises.  -to improve communication with communities  -to strengthen outreach activities | **Vaccination**:  **Full immunisation** among children aged 12–23 months shows effective result in per-protocol analysis but intention-to-treat analysis produces insignificant results | - |
| 89 | Morris et al., 2004 | Programa de asignación familiar (family allowance programme) – 1990 | Honduras;  Low socio-economic conditions | AB1. Material/ monetary incentives for caregivers;  AA2. Short-term sensitization and education campaigns; BA2. Community health worker training and education; BF1. Health system strategic planning | Motivation by providing cash transfers to mothers.  Strengthening health services would alleviate resource shortage and improve user experiences. | **Vaccination**: DPT1 improved | Intervention has been implemented in the municipalities with highest proportion of malnourishment and socio-economically deprived people hence, findings may not be applicable to other regions |
| 90 | Murthy et al., 2019 | mHealth Voice Message Service (mMitra) - 2015 | **India:** Maharashtra  -Low income | AB3. Automated voice messages to caregivers | -women receive educational messages on their phone | **Drivers:**  -Intervention reduced barriers to vaccination i.e., women in the intervention group had increased odds of knowing that the baby needs to be given vaccines  **Vaccination**:  Improved full immunisation coverage | - |
| 91 | Nagar et al., 2018 | A digital pendant and voice reminder platform (Khushi baby) - 2015 | **India:** Rajasthan  -Low education, rural | AB3. Automated voice messages to caregivers;  BG1. New HMIS or Dashboard systems (incl. improved data collection)  [Three arm study i.e., sticker only, pendant only, and pendant + reminders] | -digital pendant is a retainable health record that accounts for the broader socioecological determinants of vaccination behaviour  -collect and track immunisation records of children  -mobile application provides necessary in field decision making support for the health worker  -efficiency of health worker improvement through digitization  -tracking of performance  -voice calls sent through the dashboard may spread awareness | **Drivers:**  Intervention failed to improve barriers to vaccination i.e., increased outdegree (a proxy for centrality) of mothers corresponded with a drop in vaccination adherence  **Vaccination**:  No effect on **DPT3 completion** after 2 camps, 180 days of birth.  However, DPT3 completion after 2 months after registration was higher in pendant and pendant + reminder arms compared to control. | Udaipur was a high focus district with low immunisation rates. Further, the reasons for low immunisation may be context specific hence the results may not be generalisable to other regions.  Intervention was implemented in villages served by Seva Mandir, HTR regions of Udaipur. The findings can be applied to other rural parts of Udaipur.  However, the intervention, Khushi Baby pendant, can be generalised, especially in Rajasthan and north India where it is common among people to wear amulets |
| 92 | Nagar et al., 2020 | A digital pendant and voice reminder platform (Khushi baby) - 2017 | **India:** Rajasthan  -low income,  -low education, Rural | AB3. Automated voice messages to caregivers;  BG1. New HMIS or Dashboard systems (incl. improved data collection) | -ensure an interface between CHWs, caregiver and child.  -streamline data collection  -collect and track immunisation records of children  -enable better planning and clinical decision-making on the part of the CHWs  -improve communication between the ANM and ASHA for care coordination  -optimise management of limited health worker resources.  -identify high-risk and dropout beneficiaries  -camp reminders to beneficiaries  -better educate and remind beneficiaries about the importance of immunisation  -change the culture of action and accountability among health workers and health officials.  -ANMs use the groups to report back on high-risk patients  -provide ANMs with training, in-field support & assistance in following up on high-risk and dropout beneficiaries | **Drivers:**  -Intervention reduced barriers to vaccination i.e., more mothers in the treatment group felt empowered to make decisions about their healthcare  -Confidence in carrying out camp procedures by ANMs were not statistically significant for ability to communicate with mothers; ability to record data into the RCH register; decision-making for medication administration; decision-making for camp-based tests; and decision-making for vaccine administration  -no statistically significant difference between time spent per camp among ANMs  **Vaccination**:  **Full immunisation coverage** improved | The findings can be generalised to Gogunda, Jhadol, Lasadiya, Salumbar and Sarada parts of Udaipur. The findings of this study influenced the policy of Udaipur district to scale the Khushi Baby system to the entire district.  The findings can be generalised to other areas across India that share similar cultural beliefs in the black thread and with populations that share similar demographics. For example, people living on INR less than 1000/month, have high mobile phone access, low literacy, predominantly agricultural labour as the primary source of income, reside within 5 kilometres of a health session camp, and have low baseline full immunisation rates and MNCH awareness. |
| 93 | Nasir et al., 2017 | Mother class at the community by midwives **-** 2011 | **Indonesia**: | AA2. Short-term sensitisation & education campaign | Knowledge and awareness | **Drivers:**  Insignificant results seen on mother’s knowledge on importance of vaccination  **Vaccination**  Hep B vaccination at Birth did not improve. | - |
| 94 | Nzioki et al., 2017 | Community health strategy through CHWs – 2006 | Mwingi, Kenya | DA1. Collaborating with whole community; BF1. Health system strategic planning; BA1. Formal health worker training and education; BA2. Community health worker training and education |  | **Vaccination**: Immunization outcomes improved. | - |
| 95 | Oche et al., 2011 | Sensitisation & mobilisation of mothers **-** 2009 | **Nigeria:** | AA1. Sustained sensitization and education campaigns;  BB7. Home visits,  DA1. Collaborating with whole community |  | **Drivers:**  Improvement in knowledge but not statistically significant  **Vaccination**:  DPT1, DPT3 and dropouts from DPT1 to DPT3 coverage improved | - |
| 96 | Okeke et al., 2017 | Midwife service scheme **-** 2009 | **Nigeria:** | AA3. Public information campaign;  BA1. Formal Health worker training & education;  BE1. Building and upgrading health clinics;  BF1. Health system strategic planning | Improve access to skilled care, healthcare quality and knowledge and attitudes towards care seeking. All of these in turn increase demand for services | **Drivers:**  Improved availability of providers  **Vaccination**:  Failed to produce statistically significant result for DPT3, OPV3, Measles and BCG coverage | Restricts generalising the findings |
| 97 | Okoli et al., 2014 | SURE- P: A CCT **-** 2013 | **Nigeria:**  -Rural | AB1. Material or monetary incentives for caregivers | Parental motivation (improve safety nets) | **Vaccination**:  Failed to produce statistically significant result for OPV1 | - |
| 98 | Olayo et al., 2014 | Community Health strategy – 2010 | Butere, Mumias, Kisumu, and Garissa districts of Kenya | DA1. Collaborating with whole community; BF1. Health system strategic planning; BA2. Community health worker training and education | improving individual productivity to reduce poverty, hunger, and reduce child and maternal deaths, as well as improve education | **Vaccination**: Measles immunization improved. No changes in DPT1 and 3. | - |
| 99 | Olken  et al., 2014 | Program Keluarga Harapan (PKH): A CCT program for households and PNPM Generasi: A CCT for communities **-** 2007 | **Indonesia**:  -Low income | AB1. Material or monetary incentives for caregivers; | - Parental motivation (poverty alleviation) | **Drivers:**  **-**Demand-side: proportion of children under three years of age with health cards increased, knowledge and awareness of importance of MCH services improved  -Supply side: Program PNPM Generasi improved infrastructure, services and provider performance, however, the changes were also seen in control groups. In some areas program did not improve services such as vaccine stocks and infrastructure; door-to-door services visits by village health post cadres did not improve; availability of midwives in HTR health facility did not improve, establishing new health facility in HTR region did not improve; and lack of adequate incentives for the cadres were reported  **Vaccination:** Not measured | - |
| 100 | Owais et al., 2011 | Educational intervention **-** 2008 | **Pakistan:**  -Conflict affected, Low income, Low education, Climatic events | AB4. Written or pictorial messages (SMS, stickers, flyers etc.) to caregivers |  | **Vaccination**:  DPT3 coverage improved | - |
| 101 | Oyo-Ita et al., 2020 | Engaging traditional and religious leaders **-** 2017 | **Nigeria:** | AA1. Sustained Sensitization and education campaigns;  BA2. Community health worker training and education;  BF1. Health system strategic planning;  CA1. Faith-based outreach or outreach using local leaders; DA1. Collaborating with whole community | Creating a sense of ownership among the leaders and to promote the active participation of communities; training traditional and religious leaders improves positive influence on community attitude and foster interaction  Improvement in knowledge and monitor vaccination activities within community and act as gatekeepers | **Drivers:**  Increase in seeing immunisation cards;  Qualitative data suggested that there were more interests seen among traditional & religious leaders about immunisation, who helped in making access easier (e.g., transport) and maintaining cold chain; they also helped in sharing the information about immunisation to the community  **Vaccination**:  Failed to produce statistically significant results for full immunisation coverage.  Improved vaccination coverage (at least one vaccination) | The intervention is feasible to be implemented in LMICs where traditional and religious leaders are key influencers in the community |
| 102 | Pandey et al., 2007 | Information campaign – 2004 | Uttar Pradesh, India, rural poor | AA2. Short-term sensitization and education campaigns | Think and feel | **Vaccination**: Improved vaccination rates (at least one immunization) | - |
| 103 | Pathak & Macours 2016 | Women’s Political Reservation - 1995 | **India:** Andhra Pradesh | BF3. Changes to broader governance systems (beyond health systems) | -female leaders may favour policies beneficial to human capital, they could increase investments in education and health care infrastructure or vaccination programs | **Vaccination**:  *Children in utero during 95-01 reservation i.e., 5-6 years of age*:  -Measles, OPV coverage improved  -No effect on BCG  *Children* *born after 95-01 reservation - aged 6 to 11 months*:  -no significant impacts on Measles, OPV, BCG | Young Lives Survey dataset for Andhra Pradesh covering 20 mandals in 6 rural districts (Srikakulam and West Godavari in the Coastal region; Anantapur and Cuddapah in Rayalaseema; & Mahboobnager and Karimnaga in Telangana) |
| 104 | Powell-  Jackson et al., 2018 | Face-to-face health education to mothers - 2015 | **India:** Uttar Pradesh | AA2. Short-term sensitization and education campaigns | -Information provided to mothers on the benefits of the vaccine | **Drivers:**  -Intervention reduced barriers to vaccination i.e., knowledge about child vaccination. Intervention had an effect on perception of mothers about DPT vaccine in women who had perceptions of efficacy below 50% at baseline  -Intervention failed to improve barriers to vaccination i.e., no statistically significant difference was observed about perceptions of vaccine efficacy (intervention did not have an effect on mothers who were already convinced of the efficacy of the DPT vaccine—i.e., those who had perceptions of efficacy above 50% at baseline)  **Vaccination**:  **-****DPT3**, full immunisation, measles coverage improved  -No improvement seen in BCG coverage | There were low levels of mothers’ knowledge and perceptions of efficacy of vaccines at baseline, however, the six study districts were not the worst performing districts of the state in terms of vaccination coverage.  -In areas with low immunisation rates, the intervention may produce large effects.  -In areas with high baseline awareness and knowledge levels of mothers, the intervention may be less effective  -A local organisation implemented the intervention, to scale up and long-term sustainability relying on government would be required |
| 105 | Pramanik et al., 2020 | Stimulate, Appreciate, Learn and Transfer community engagement approach - 2017 | **India:** Assam  -Rural region | AA1. Sustained sensitization and education campaigns;  DA1. Collaborating with whole community;  BB7. Home visits | -community identifies a problem, makes an action plan, takes action and learns from the process  -trained facilitators conducting home visits in villages for community engagement  -transfer of knowledge and experience | **Drivers:**  -Intervention reduced barriers to vaccination i.e., qualitative findings demonstrated improvement in immunisation knowledge and understanding the details such as the immunisation schedule and names of the vaccine-preventable diseases  -mothers and mothers-in-law were generally more aware about the importance of immunisation  -a reduction in fear regarding side effects following vaccination was reported  -improved attitudes towards immunisation in some target areas by addressing misconceptions about vaccines  -Intervention failed to improve barriers to vaccination i.e., vaccination card availability  **Vaccination**:  No effect on **full immunisation** (12-23 months), DPT3 (6-23 months) | - |
| 106 | Prinja et al., 2017 | m-health application used by community health volunteers - 2011 | **India:** Uttar Pradesh  -Low education  -Rural region | BG1. New HMIS or Dashboard systems (incl. improved data collection) | -job aid for ASHAs  -Improved knowledge about the need for services  -continuous monitoring and supervision of ASHA performance through generation of real-time data on utilisation of services  -training of ASHAs- skills | **Drivers:**  -Intervention reduced barriers to vaccination i.e., coverage of ASHA and their performance improved  **Vaccination**:  -Statistically insignificant results for Full immunisation coverage | - |
| 107 | Rahman & Pallikadavath, 2019 | A Conditional Cash Transfer programme: Janani Suraksha Yojana - 2005 | **India:** Multiple states | AB1. Material or monetary incentives for caregivers;  BD1. Material or monetary incentives for health workers | -to increase knowledge and improve practices  - to improve provision of quality of services  -to increase availability and access to a quality continuum of services  -to increase informed demand for, and utilisation of services  -to increase participation, accountability, and responsiveness to communities’ voices in MCH services | **Vaccination**:  Improved OPV1 (within 2 weeks of birth), BCG, Measles, DPT, OPV. | Considered the data from DLHS 4- Survey took place in low focus states (not nationally representative unlike DLHS 2 and 3) |
| 108 | Rahman et al., 2016 | Improving Maternal Neonatal Child Survival (IMNCS) program - 2008 | **Bangladesh:**  -Low income | AA1. Sustained sensitisation & education campaigns;  BA2. Community health worker training & education;  BF1. Health system strategic planning;  DA1. Collaborating with whole community | -knowledge and practices related to MNCH services  - provision of quality MNCH services at the community and facility level | **Vaccination**:  Full immunisation improved but effect was not statistically significant | - |
| 109 | Rahman et al., 2008 | Thinking health programme- Cognitive behaviour therapy-based intervention for depressed perinatal women delivered by lady health workers – 2005-06 | Gujar Khan and Kallar Syedan in Pakistan; Rural | BF1. Health system strategic planning; BA2. Community health worker training and education | Treating the depression | **Vaccination**: Improved full immunization | - |
| 110 | Rao, 2014 | Community Health Worker (Accredited Social Health Activists) Program -2005 | **India:** Multiple states | BA2. Community health worker training and education;  BF1. Health system strategic planning | -ASHA worker to counsel women on the importance of immunisation and to mobilise | **Drivers:**  -Intervention reduced barriers to vaccination i.e., improvement in outreach  -health workers motivating caregivers for immunisation  -improvement in awareness about the need for vaccination among mothers  -Improvement in mother’s knowledge about the place of immunisation.  **Vaccination**:  **Full immunisation,** BCG, DPT3, OPV3, Measles coverage improved | Considered the data from DLHS- Survey took place in 34 states and union territories in India (excluding Nagaland) |
| 111 | Rasella et al., 2013 | The Bolsa Família Program: A CCT program **-** 2003 | **Brazil:**  -Low income | AB1. Material or monetary incentives for caregivers | Parental motivation (poverty alleviation) | **Vaccination**:  Measles, polio, and DPT vaccine coverage over 95% among children younger than 1 year increased | - |
| 112 | Robertson et al., 2013 | unconditional and conditional cash transfers – 2009-10 | Manicaland, Zimbabwe | AB1. Material/ monetary incentives for caregivers | Cash transfers provides resources such as transport cost and reduces children to work | **Vaccination**: Increased full vaccination but not significant in both groups compared to control | - |
| 113 | Robinson et al., 2001 | On-the-job peer training of nurses – 1993 | Indonesia | BA1. Formal health worker training and education | Healthcare experiences | **Drivers:** quality of immunization practices improved (adherence to cold-chain protocols, use of sterilization, reporting, administering immunizations to sick children, and in using more energetic, problem solving approaches to track and vaccinate children in villages)  **Vaccination**: DPT1, OPV3, measles coverage improved. | Context dependent |
| 114 | Roy et al., 2008 | Rural Maintenance Programme (RMP) – 1983 | Bangladesh, destitute women | BF3. Changes to broader governance systems (beyond health systems); AA2. Short-term sensitization and education campaigns | Skill training would make the women self-reliant and education package would improve nutritional status and health related knowledge and practices | **Vaccination**: Improved immunization outcomes but not significantly. | - |
| 115 | Rusa et al., 2009 | Performance-based financing – 2005 | Rural districts (Ruli, Rutongo, Kabgayi, Kabuga & Bugesera) and semirural district (Muhima) of Rwanda | BD5. Pay-for-performance schemes | - | **Drivers:** Quality of services such as cold chain maintenance, vaccination record keeping increased but fully vaccinated children/month was not improved. | - |
| 116 | Ryman et al., 2011 | Reaching Every District (RED) strategy - 2005 | **India:** Assam  -Low income, rural region | BB7. Home visits;  BF1. Health system strategic planning;  DA1. Collaborating with whole community BG1. New HMIS_Dashboard systems (incl. improved data collection) | -strengthening core sub-national routine vaccination program functions: re-establishing outreach services; providing supportive supervision; monitoring and using data for action; improving planning and resource management; and increasing community links with service delivery | **Drivers:**  -Intervention reduced barriers to vaccination i.e., quality improvements at health facility  **Vaccination**:  No difference in **full immunisation** between intervention and comparison districts | - |
| 117 | Saggurti et al., 2018 | Health intervention integration within women’s self-help groups on collectivization and healthy practices (Part of Ananya Program) - 2011 | **India:** Bihar  -Low education, rural | AA2. Short-term sensitization and education campaigns | -increase availability of health services  -improve the quality of family health services: strengthen quality of care protocols and their application at the primary health centre and to provide continual training to CHWs to increase the number and quality of interactions between families and CHWs | **Drivers:**  -Intervention reduced barriers to vaccination i.e., performance of ASHA and AWWs (improved enumeration and mapping, frequency of home visits)  -Intervention failed to improve barriers to vaccination i.e.,  -Availability of immunisation card improved during first phase however, in second phase no statistically significant result was observed  -health workers reminding mothers of the next vaccination declined in second phase  **Vaccination**:  Improved age-appropriate immunisation | - |
| 118 | Sankar, 2013 | Community Mobilisation and Integrated Planning (Bachpan Program) - 2006 | **India:** Madhya Pradesh  -Low income, rural tribal | AA1. Sustained sensitization and education campaigns;  BA2. Community health worker training and education;  BF1. Health system strategic planning;  DA1. Collaborating with whole community | -mobilisation of the village community; this in turn can lead to better interaction within the community and between the providers and beneficiaries, and improve awareness about child development issues | **Drivers:**  -Intervention reduced barriers to vaccination, i.e., improvement activities at Anganwadi centres improved parents accessing the services.  **Vaccination**:  Full immunisation, DPT1, 2, 3, OPV0, 1, 2, 3, Measles coverage improved (1-2 years of age).  No effect on BCG coverage | - |
| 119 | Sato & Belel, 2020 | Performance-based financing **-** 2015 | **Nigeria:**  -Low education | BD5. Pay-for-performance scheme; BF4. Health system financing | Improvement in healthcare service quality | **Drivers:**  Seeing immunisation cards improved; improvement in knowledge about measles timing improved  Knowledge about immunisation schedule  **Vaccination**:  Improved BCG, measles, and full immunisation services.  Failed to produce statistically significant result for OPV and DPT services | Sample is not nationally representative hence findings cannot be generalised |
| 120 | Sengupta et al., 2017 | Community-based intervention - 2013 | **India:** Punjab  -Low income, slums | BB6. Outreach to migrant populations;  DB1. Community tracking and registering | -availability of outreach immunisation clinics and community guardians so that the access to immunisation would increase | **Drivers:**  -Intervention reduced barriers to vaccination i.e., qualitative evidence shows that mothers were more aware and cooperative  **Vaccination**:  **Full immunisation** by 1 year of age improved | - |
| 121 | Seth et al., 2018 | Mobile phone incentives - 2016 | **India:** Haryana | AB1. Material or monetary incentives for caregivers;  AB4. Written or pictorial messages (SMS, stickers, flyers etc.) to caregivers  BG1. New HMIS/Dashboard systems (incl. improved data collection) | -motivation to caregivers via reminders and incentives | **Vaccination**:  Automated reminders did not produce statistically significant result for **vaccination coverage (unspecified).**  Automated reminders with compliance linked incentives improved **vaccination coverage (unspecified).** | Intervention can be applied to low resource settings |
| 122 | Shei et al., 2014 | The Bolsa Família Program: A CCT program **-** 2003 | **Brazil:**  -Low income,  Slum dwellers | AB1. Material or monetary incentives for caregivers | Parental motivation (poverty alleviation) | **Vaccination**:  Increased vaccination coverage (unspecified) | Lack of generatability as study was restricted to slum community in north-eastern Brazil |
| 123 | Sherry et al., 2017 | Pay-for-performance **-** 2006 | **Rwanda:** | BD5. Pay-for-performance scheme | Improve quality of health services | **Vaccination**:  Failed to improve full immunisation coverage | - |
| 124 | Shukla, 2018 | Health Governance Intervention – 2012 | Afghanistan, conflict affected | BF1. Health system strategic planning; DA1. Collaborating with whole community | Governance could increase accountability and quality of services | **Vaccination**: Improved penta3 coverage | Lack of |
| 125 | Siddiqi et al., 2020 | Vaccine reminder and tracker silicon bracelet **-** 2017 | **Pakistan:**  -Low education | AB2. Non material incentives for caregivers; AB4. Written or pictorial messages (SMS, stickers, flyers etc.) to caregivers | Bracelet would serve as a visible and durable reminder even for uneducated mothers | **Vaccination**:  DPT3, Measles coverage and timeliness of vaccination were not improved | - |
| 126 | Soeters et al., 2011 | Performance based ﬁnancing – 2005 | Katana and Idjwi districts, Democratic Republic of Congo (DRC); poor | BD5. Pay-for-performance schemes | Improves motivation and thereby performance | **Drivers:** Some of the intermediate outcomes improved.  **Vaccination**: Improved vaccination but not significant | - |
| 127 | Tandon & Sahai, 1988 | Integrated Child Development Services Scheme - 1975 | India | BF1. Health system strategic planning; DA2. Collaborating with selected community groups and networks; DB1. Community tracking and registering |  | **Vaccination**: Improved immunization outcomes | - |
| 128 | Uddin et al., 2016 | Mobile phone intervention to improve immunisation **-** 2013 | **Bangladesh**:  -Low income, rural HTR areas (with most wetland) & children living in urban streets | AB4. Written or pictorial messages (SMS, stickers, flyers etc.) to caregivers;  BD4. Written or pictorial messages (SMS, stickers, flyers etc.) to health workers;  BG1. New HMIS or dashboard system (incl. improved data collection) | -Reminding mothers  -Improvement of data collection and tracking | **Vaccination**  Full immunisation and age-appropriate vaccination improved | Findings are generalizable to other HTR remote, rural & slum areas in Bangladesh provided full vaccination coverage is around 42–70% and mobile phone ownership is 75–89% |
| 129 | Unger, 1991 | Intensive immunization campaign – 1986-87 | Thies Health District, Senegal | BC1. National/sub-national immunisation days; BA1. Formal health worker training and education; BF1. Health system strategic planning |  | **Drivers**: No effect BCG coverage, on completeness of immunization, filling immunization data in card, communication between caregiver and healthcare provider and cold chain management | - |
| 130 | Usman et al., 2011 | Reminder type immunisation card and centre-based information and motivation session **-** 2005 | **Pakistan:**  -Conflict affected, Low education, Low income, climatic events | AA2. Short-term sensitization and education campaigns; AB4. Written or pictorial messages (SMS, stickers, flyers etc.) to caregivers | Parental motivation and improving awareness | **Vaccination**:  DPT 3 coverage improved | - |
| 131 | Vaidyanathan, 2019 | Education and communication strategy - 2012 | **India:**  Maharashtra | AA2. Short-term sensitization and education campaigns | -IEC on immunisation to improve the knowledge of immunisation | **Drivers:**  -Intervention reduced barriers to vaccination i.e., knowledge about child immunisation improved  **Vaccination**:  Full and partial immunisation coverage improved Reduction in un-immunisation. | - |
| 132 | Varghese et al., 2014 | The Yashoda program (provision of facility-based support worker or birth companion) - 2008 | **India:** Rajasthan, Odisha  -Rural areas | AB2. Non-material incentives for caregivers;  BA2. Community health worker training and education;  BD1. Material or monetary incentives for health workers;  BF1. Health system strategic planning | -Yashoda process provides a quick response to help the hospital system to cope with the increasing demand for quality care for the new born by having a dedicated team to take on non-clinical tasks and free up the time of the nurses to focus on curative tasks.  -Orient the mother about basic newborn care and immunisation | **Vaccination**:  No effect on BCG and OPV0 coverage | - |
| 133 | Wagstaff and Yu, 2007 | Basic Health Service Project (health sector reform project) - 1998 | Gansu province, China, poor | BF1. Health system strategic planning; BA1. Formal health worker training and education; BE1. Building & upgrading health clinics; AB5. Changes to health system user fees | Healthcare experiences | **Vaccination**: No impact on immunization outcomes |  |
| 134 | Wadhwa, 2019 | Conditional Cash Transfers and Parental Investment in Daughters - 2005 | **India:** Haryana  Low income, low education | AB1. Material or monetary incentives for caregivers | Motivation to caregivers | **Vaccination**:  Improved **vaccination coverage** | - |
| 135 | Wallace et al., 2019 | Parental home-based reminders and stickers **-** 2016 | **Indonesia** | AB4. Written or pictorial messages (SMS, stickers, flyers etc.) to caregivers | Parental reminders | **Vaccination:**  Children in the HBR + sticker group were 50% more likely to have received a DTPcv3 vaccination within 60 days of DTPcv1 vaccination | - |
| 136 | Wang et al., 2015 | Integrating early infant HIV diagnosis with the expanded programme on immunization – 2013 | Livingstone, Monze, and Choma districts in the Southern Province of Zambia | BF1. Health system strategic planning;  BA1. Formal health worker training and education |  | **Vaccination**: Improved DPT1 but not significant | - |
| 137 | Webster et al., 2019 | Community Engagement Strategy (Fifth child intervention) – 2015 | Kitgum, Lamwo, and Agago districts, Uganda | DA1. Collaborating with whole community; DB1. Community tracking and registering; BB5. Outreach to vulnerable populations (hard-to-reach, SES, caste, etc.); BF1. Health system strategic planning; BA1. Formal health worker training and education. | Improvement in defaulter tracing would increase immunization coverage in hard-to-reach region | **Drivers**: Accessibility of immunization services improved  **Vaccination**: No impact on DPT3, measles, timeliness, dropouts. | - |
| 138 | Weldemariam, 2010 | Fiscal decentralisation **-** 2002 | **Ethiopia:** | EB1. Non-health or education infrastructure | - | **Vaccination**:  Measles, DPT3 and full immunisation coverage improved | - |
| 139 | Wong et al., 2019 | Measles immunisation campaigns - 2008 | **India:** Multiple states | BC1. National or sub-national immunisation days;  BF2. Vaccination guidelines | Improving outreach | **Vaccination**: Not measured | Data from Million Death study |
| 140 | Younes et al., 2014 | Participatory women’s groups **-** 2010 | **Bangladesh:** -Climatic events- floods | AA1. Sustained sensitization and education campaigns;  DA2. Collaborating with selected community groups & networks | - | **Vaccination**  Full immunisation was not improved | - |
| 141 | Zang et al., 2015 | Performance based ﬁnancing – 2012 | Littoral, Cameroon, urban | BD5. Pay-for-performance schemes | improved staff motivation | **Drivers:** improved service quality  **Vaccination**: Improved immunization | - |
| 142 | Zeng et al., 2018 | Result-based financing – 2012 | Niari, Plateaux and Pool departments in DRC; Rural | BD5. Pay-for-performance schemes | Increased autonomy, strengthened data reporting, and enhanced capacity to manage health facilities, thereby boost motivation to increase coverage and quality of services | **Drivers:** Improved perceived quality of care.  **Vaccination**: DPT3 coverage did not improve (it statistically decreased). No impact on BCG & full immunization. | - |

## **Figure 3A: Countries of included impact evaluations**

Note: the total no of impact evaluations in this figure are 146 as three studies (Barham et al., 2007; Bradley & Igras, 2005; Lee, 2015) were conducted in more than one country.

# **Appendix 4: Multicomponent interventions addressing behavioral and social drivers of vaccination and their impact on immunization**

| **Impact evaluations​** | **Barriers addressed** | | | | **Intervention components** | | | | | **Intervention effect as reported by studies** |
| --- | --- | --- | --- | --- | --- | --- | --- | --- | --- | --- |
|  | **Think & feel** | **Social processes** | **Motivation** | **Practical barriers** |  |  |  |  |  |  |
| 1. **Interventions addressing attitudes and knowledge about immunization** | | | | | | | | | | |
| Brugha & Kavany, 1996 | Yes |  |  | Yes | Short-term sensitization and education campaigns |  |  | Home visits |  | Improved immunization outcomes |
| Gurley et al., 2020 | Yes | - | - | Yes | Short-term sensitization & education campaigns |  |  | Outreach to vulnerable populations | Community health worker training & education | Insignificant effect on full immunization & timeliness. Improved DPT1-3 dropouts and DPT3 coverage and other intermediate outcomes |
| Bolam et al., 1998 | Yes |  |  | Yes | Short-term sensitization & education campaigns |  |  |  | Community health worker training & education | Insignificant effect on immunization outcomes |
| Johri et al., 2020 | Yes | Yes | Yes | Yes | Short-term sensitization & education campaigns | Public information campaigns | Automated voice messages to caregivers | Home visits | Collaborating with whole community | Improved immunization outcomes |
| Usman et al., 2011 | Yes | - | Yes | Yes | Short-term sensitization & education campaign |  | Written or pictorial messages to caregivers |  |  | Improved immunization outcomes |
| Banwat et al., 2015 | Yes | - | - | Yes | Sustained sensitization & education campaigns |  |  | Home visits | Community tracking & registering | Insignificant effect on full immunization but improved caregiver knowledge |
| Goel et al., 2011 | Yes | - | Yes | Yes | Sustained sensitization & education campaigns | Public information campaigns | Material or monetary incentives and training & education for health workers | Outreach to vulnerable population and Home visits | Health system strategic planning | Improved immunization outcomes |
| Hutchison et al., 2006 | Yes |  |  | Yes | Public information campaigns | Written or pictorial messages to caregivers |  |  |  | Improved immunization outcomes |
| **2) Behavior linked incentivized interventions for caregivers** | | | | | | | | | | |
| Morris et al., 2004 | Yes |  | Yes | Yes | Monetary incentives for caregivers | Short-term sensitization and education campaigns |  | Community health worker training and education | Health system strategic planning | Improved immunization outcomes |
| Carvalho et al., 2014; Rahman & Pallikadavath, 2019 | - | - | Yes | Yes | Material or monetary incentives for caregivers ​ |  | Material or monetary incentives for health workers​ |  | ​ | Improved immunization outcomes |
| Goodson et al., 2012 |  |  | Yes | Yes | Material incentives for caregivers |  |  |  | National/sub-national immunization days | Improved immunization outcomes |
| Banerjee et al., 2010 | **-** | **-** | Yes | Yes | Material or monetary incentives for caregivers ​ |  |  | Outreach to vulnerable  populations | -​ | Improved immunization outcomes |
| Seth et al., 2018 | - | - | Yes | Yes | Material or monetary incentives for caregivers | Written or pictorial messages to caregivers |  |  | New HMIS/Dashboard systems | Improved immunization outcomes |
| Demilew et al., 2021 | **-** | **-** | Yes | Yes | Non-monetary incentives for caregivers |  | Health worker involvement in planning & monitoring | Written or pictorial messages for health workers | Paper-based tracking | Insignificant effect on immunization outcomes |
| Roy et al., 2008 | Yes |  | Yes |  | Changes to broader governance systems (beyond health systems) | Short-term sensitization and education campaigns |  |  |  | Improved immunization outcomes but not significant |
| **3) Interventions addressing practical constraints faced by caregivers** | | | | | | | | | | |
| Hagiwara et al., 2012 | Yes |  |  | Yes | Written or pictorial messages to caregivers | Short-term sensitization and education campaigns | Formal health worker training and education |  |  | No impact on immunization outcomes |
| Brown et al., 2016 | - | - | Yes | Yes | Automated voice messages to caregivers |  | Health worker training & education |  |  | Improved immunization outcomes |
| **4) Interventions addressing healthcare service quality** | | | | | | | | | | |
| Wong et al., 2019 | - | - | - | Yes | National or sub-national immunization days | Vaccination guidelines |  |  |  | - |
| Sengupta et al., 2017 | Yes | - | Yes | Yes |  | Outreach to migrant populations​ |  |  | Community tracking and registering | Improved immunization outcomes |
| Atnafu et al., 2017 | - | - | - | Yes | New HMIS or dashboard systems |  |  | Capacity building for existing systems |  | Insignificant effect on full immunization |
| Chen et al., 2016 |  |  |  | Yes | New HMIS/Dashboard systems | Written or pictorial & automated voice messages to caregivers |  |  |  | Insignificant effect on immunization outcomes |
| Dissieka et al., 2019 |  |  |  | Yes | New HMIS/Dashboard systems | Written or pictorial & automated voice messages to caregivers |  |  |  | Improved immunization outcomes |
| Busso et al., 2015 | Yes | - | Yes | Yes | New HMIS or dashboard systems | Home visits |  |  |  | Improvement in knowledge and self-reported practice |
| Modi et al., 2019 | Yes | - | Yes | Yes | New HMIS or Dashboard systems | Written or pictorial messages to caregivers | Automated voice & written or pictorial messages to health workers | Capacity building (e.g., training) for existing systems |  | Insignificant effect on DPT3 |
| Nagar et al., 2020 | Yes | - | - | Yes | New HMIS or Dashboard systems ​ | Automated voice messages to caregivers |  | ​ |  | Improved full vaccination and other intermediate outcomes |
| Nagar et al., 2018 | Yes | - | - | Yes | New HMIS or Dashboard systems ​ | Automated voice messages to caregivers |  |  |  | Improved DPT3 |
| Uddin et al., 2016 | - | - | - | Yes | New HMIS or Dashboard systems | Written or pictorial messages to caregivers | Written or pictorial messages to health workers |  |  | Improved immunization outcomes |
| Hategeka et al., 2019 | - | - | Yes | Yes | New HMIS or dashboard systems | Written or pictorial messages to caregivers |  | Written or pictorial messages for health workers |  | Insignificant effect on immunization outcomes |
| Djibuti et al., 2009 |  |  |  | Yes | Formal health worker training & education |  |  | Health system strategic planning |  | Improved immunization outcomes |
| Unger, 1991 |  |  |  | Yes | Formal health worker training & education | National/sub-national immunization days |  | Health system strategic planning |  | No effect on immunization and intermediate outcomes |
| Adamu et al., 2019 | - | - | - | Yes | Formal health worker training & education |  | Formal health worker involvement in planning & monitoring |  |  | Improvement in capturing missed opportunities for vaccination |
| Mazumder et al., 2014 | Yes | - | - | Yes | Formal & community health worker training and education | Home visits & National or sub-national immunization days | Pay-for-performance schemes | Health system strategic planning | Sustained sensitization & education campaigns | Insignificant effect on immunization outcomes |
| Mohan et al., 2011 | Yes | - | - | Yes | Formal & community health worker training and education | Home visits |  |  |  | Insignificant effect on full immunization |
| Rao, 2014 | Yes | - | Yes | Yes | Community health worker training & education​ |  |  | Health system strategic planning​ | -​ | Immunization and intermediate outcomes improved |
| Balasubramaniam et al., 2018 | - | - | - | Yes | Community health worker training & education |  |  | Health system strategic planning |  | Insignificant effect on DPT3 |
| Manyazewal et al., 2018 | - | - | - | Yes | Formal health worker training & education |  | Capacity building for existing systems | Health system strategic planning |  | Improved immunization outcomes except full immunization |
| Okeke et al., 2017 | Yes | - | - | Yes | Formal Health worker training & education |  | Building or upgrading health clinics | Health system strategic planning | Public information campaign | Insignificant effect on immunization outcomes |
| Carnell et al., 2014 | - | - | - | Yes | Health worker training & education |  | Non-material incentives for health workers | Health system strategic planning | New HMIS or dashboard systems | Improved immunization and intermediate outcomes |
| Varghese et al., 2014 | - | - | Yes | Yes | Community health worker training and education | Non-material incentives for caregivers | Material or monetary incentives for health workers | Health system strategic planning |  | Insignificant effect on immunization outcomes |
| Borkum et al., 2014 | - | - | - | Yes |  |  | Material or monetary incentives for health workers | Non-material incentives for health workers |  | Insignificant effect on immunization outcomes |
| Sato & Belel, 2020 | - | - | - | Yes |  |  | Pay-for-performance scheme | Health system financing |  | Improved BCG, measles, full immunization & other intermediate outcomes. Insignificant effect on OPV & DPT |
| Arifeen et al., 2009 | Yes | Yes |  | Yes | Health system strategic planning | Health worker training & education | Faith-based outreach/outreach using local leaders | Collaborating with selected community groups and networks | Sustained sensitization and education campaigns | No improvement in immunization outcomes |
| Tandon & Sahai, 1988 |  |  | Yes | Yes | Health system strategic planning |  |  | Collaborating with selected community groups and networks | Community tracking and registering | Improved immunization outcomes |
| Wagstaff & Yu, 2007 |  |  |  | Yes | Health system strategic planning | Formal health worker training & education | Changes to health system user fees | Building & upgrading health clinics |  |  |
| Admassie et al., 2009 |  |  |  | Yes | Health system strategic planning | Community health worker training & education |  | Building & upgrading health clinics | Home visits | Improved immunization outcomes |
| Assegaai et al., 2018 |  |  |  | Yes | Health system strategic planning | Community health worker training & education |  |  |  | Few immunization-related outcomes improved |
| Biemba et al., 2016 |  |  |  | Yes | Health system strategic planning | Community health worker training & education |  |  |  | Improved full immunization |
| Bradley & Igras, 2005 |  |  |  | Yes | Health system strategic planning | Formal health worker training & education |  |  |  | Improved intermediate outcomes |
| Drain et al., 2006 |  |  |  | Yes | Vaccination guidelines (AD syringes) | Formal health worker training & education | Health system strategic planning |  |  | Improved outcomes |
| Wang et al., 2015 |  |  |  | Yes | Health system strategic planning | Formal health worker training & education |  |  |  | No impact on immunization outcomes |
| Rahman et al., 2008 |  |  |  | Yes | Health system strategic planning | Community health worker training & education |  |  |  | Improved full immunization |
| **5) Intervention strategies used to mobilize communities and influence social norms** | | | | | | | | | | |
| Björkman & Svensson, 2009 | Yes |  |  | Yes | Collaborating with whole community |  |  |  | Health system strategic planning | Improved outcomes |
| Alhassan et al., 2019 |  |  |  | Yes | Collaborating with whole community |  |  |  | Health system strategic planning | Improved immunization outcome |
| Shukla, 2018 |  | Yes |  | Yes | Collaborating with whole community |  |  |  | Health system strategic planning | Improved DPT3 |
| Olayo et al., 2014 |  | Yes |  | Yes | Collaborating with whole community |  | Community health worker training & education |  | Health system strategic planning | Measles improved but not DPT |
| Nzioki et al., 2017 | Yes | Yes |  | Yes | Collaborating with whole community |  | Health worker training & education | Home visits | Health system strategic planning | Immunization outcomes improved |
| Webster et al., 2019 |  | Yes |  | Yes | Collaborating with whole community | Community tracking and registering | Formal health worker training & education | Outreach to vulnerable population | Health system strategic planning | No impact on immunization outcomes |
| Findley et al., 2013 | Yes | Yes | - | Yes | Collaborating with whole community | Sustained sensitization & education campaigns; Public information campaign | Health worker training & education |  | Health system strategic planning | Improved immunization outcomes |
| Oche et al., 2011 | Yes | Yes | - | Yes | Collaborating with whole community | Sustained sensitization & education campaigns |  | Home visits |  | Improved immunization outcomes |
| Cristia et al., 2011; Cristia, Prado, Peluffo, 2015; Cristia, Evans, Kim, 2015 | Yes | Yes | - | Yes | Collaborating with whole community |  |  | Outreach to vulnerable populations | Health system strategic planning | Improved immunization outcomes |
| Ryman et al., 2011 | - | Yes | - | Yes | Collaborating with whole community |  | New HMIS or Dashboard systems | Home visits | Health system strategic planning | Insignificant effect on immunization but healthcare quality improved |
| Andersson et al., 2009 | Yes | Yes |  |  | Collaborating with selected community groups and networks | Short-term sensitization and education campaigns |  |  |  | Improved immunization and intermediate outcomes |
| Janssens, 2011 | Yes | Yes | Yes | - | Collaborating with select community groups & networks​ | Sustained sensitization & education campaigns​ |  | -​ |  | Improved immunization and intermediate outcomes |
| Younes et al., 2014 | Yes | Yes | - | - | Collaborating with selected community groups & networks | Sustained sensitization & education campaigns |  |  |  | Insignificant effect on full immunization |
| Oyo-Ita et al., 2020 | Yes | Yes | Yes | Yes | Collaborating with whole community | Faith-based outreach or outreach using local leaders | Community health worker training and education | Health system strategic planning | Sustained Sensitization & education campaigns | Insignificant effect on full immunization but improved partial vaccination and other intermediate outcomes |
| Habib et al., 2017 | Yes | Yes | Yes | Yes | Collaborating with selected community groups & networks | Faith-based outreach or outreach using local leaders | Community health worker training & education | Outreach to vulnerable populations |  | OPV3 & full vaccination coverage improved |
| Memon et al., 2015 | Yes | Yes | - | Yes | Collaborating with selected community groups & networks | Sustained sensitization & education campaigns | Community health worker training & education |  |  | Improved full immunization |
| Bhuiya et al., 2016 | Yes | Yes | Yes | Yes | Collaborating with whole community and selected community groups & networks | Sustained sensitization & education campaigns | Formal & community health worker training & education | Building & upgrading health clinics | Capacity building (e.g., training) for existing systems | Improved full immunization and other intermediate outcomes |
| Rahman et al., 2016 | Yes | Yes | - | Yes | Collaborating with whole community | Sustained sensitization & education campaigns | Community health worker training & education | Health system strategic planning |  | Insignificant effect on full immunization |
| Mohanan et al., 2020 | Yes | Yes | Yes | Yes | Collaborating with whole community​ |  | ​ | Home visits |  | Improved immunization outcomes except measles |
| More et al., 2017 | Yes | Yes | Yes | Yes | Collaborating with whole community |  |  | Home visits |  | Improved full immunization as per per-protocol analysis but insignificant with intension-to-treat analysis |
| Pramanik et al., 2020 | Yes | Yes | Yes | Yes | Collaborating with whole community | Sustained sensitization & education campaigns |  | Home visits |  | Insignificant effect on immunization outcomes but improved intermediate outcomes |
| Sankar, 2013 | Yes | Yes | Yes | Yes | Collaborating with whole community | Sustained sensitization & education campaigns | Community health worker training and education |  | Health system strategic planning | Improved immunization outcomes |

Note: In some of the included studies, it was not explicitly stated that the interventions addressed a particular barrier(s). We made reasonable assumptions on the barriers being addressed by considering the intervention components and causal pathways. For instance, pay-for-performance schemes can potentially improve performance of health workers thereby improving caregiver’s experiences of healthcare quality. Another example is prolonged exposure to media (public information campaigns) can potentially motivate caregivers. Furthermore, some of the strategies might also influence social processes, e.g., sensitization programs, but unless intervention included community mobilization activities or mentioned influencing the community, we did not mark them as intervention addressing social norms.

1. One-time sensitization campaign as described by Engelbert et al. (2022) has been termed as short-term sensitization campaign for the purpose of this review [↑](#footnote-ref-1)
